# Integrated transcriptomic and metabolomic analysis reveals the central role of phenylpropanoid biosynthesis in pea resistance to powdery mildew

**DOI:** 10.3389/fpls.2026.1793693

**Published:** 2026-04-22

**Authors:** Yuanli Li, Jianying Lu, Yang Shao, Chang Wang, Lijuan Zhang, Gengmei Min

**Affiliations:** 1 Institute of Biotechnology, Gansu Academy of Agricultural Sciences, Lanzhou, Gansu, China; 2 Gansu Linxia Hui Autonomous Prefecture Academy of Agricultural Sciences, Linxia, Gansu, China; 3 Institute of Crops, Gansu Academy of Agricultural Sciences, Lanzhou, Gansu, China

**Keywords:** metabolomics, pea, phenylpropanoid biosynthesis, powdery mildew, transcriptomics

## Abstract

Pea powdery mildew severely reduces crop yield and quality, yet the dynamic molecular and metabolic regulatory networks underlying resistance differences between different pea varieties remain poorly understood. Here, we profiled the defense landscape of susceptible (Longwan 3) and resistant (Longwan 5) pea varieties across three infection stages (0, 3, and 6 days post-inoculation) via integrated transcriptomic and metabolomic analyses. Multi-omics data revealed pronounced differences in metabolic reconfiguration (1754 metabolites identified) and transcriptional reprogramming (34566 genes annotated) between the two pea cultivars. Integrated Kyoto Encyclopedia of Genes and Genomes (KEGG) enrichment analyses of differentially expressed genes (DEGs) and differentially accumulated metabolites (DAMs) consistently identified the phenylpropanoid biosynthesis pathway as the core pathway driving powdery mildew resistance. The resistant variety exhibited sustained upregulation of key biosynthetic genes (e.g., phenylalanine ammonia-lyase and cinnamate 4-hydroxylase), which directly drove the accumulation of defensive metabolites including ferulic acid and sinapic acid. Although some gene-metabolite correlations were consistent, others (e.g., those involving β-glucosidase and peroxidase genes) reflected a complex, multi-layered regulatory network involving post-transcriptional regulation and metabolic feedback mechanisms. Our study advances our understanding of dynamic defense mechanisms in legumes, and offers novel molecular targets for enhancing powdery mildew resistance, as well as efficient markers for precision breeding of elite pea varieties.

## 1 Introduction

Legumes are the second most important crop group globally after cereals, playing a critical role in sustainable agriculture due to their ability to fix atmospheric nitrogen (Assen, 2020). Among legumes, pea (*Pisum sativum* L.) is widely recognized as a nutritionally valuable and agronomically adaptable crop, cultivated in more than 100 countries worldwide. Pea is rich in proteins, soluble sugars, minerals, vitamins, lipids, essential amino acids, slowly digestible starch, and dietary fiber (Santalla et al., 2001; Tzitzikas et al., 2006; Nawab et al., 2008; Smýkal et al., 2012). In China, pea has been cultivated for two millennia, and the yields of dry peas and green peas rank among the highest globally (Sun et al., 2015; Yang et al., 2022a). However, the development of the pea industry is constrained by stagnant yields caused by various biotic and abiotic stresses, including nematodes, insects, fungi, bacteria, and viruses (Khan, 2019; Ullah et al., 2020; Greveniotis et al., 2021). Among these constraints, powdery mildew, mainly caused by *Erysiphe pisi*, is particularly severe, leading to global pea yield losses of approximately 25–50% (Fondevilla et al., 2011; Sun et al., 2016), and even causing losses of more than 80% in severely infected pea varieties (Nisar and Ghafoor, 2011; Fondevilla and Rubiales, 2012). As an airborne disease, powdery mildew leads to a decrease in pod number, node number, seed number, plant height, grain yield, and plant biomass (Azmat et al., 2012; Villegas-Fernández et al., 2021; Banyal et al., 2022). Although chemical fungicides can control pea powdery mildew, their repeated use causes environmental risks and drives the development of fungicide resistance in pathogens. Therefore, the development and deployment of genetically resistant varieties represent the most sustainable and economically viable strategy for disease management.

Three major genes (two recessive genes, *er1* and *er2*, and one dominant gene *Er3*) for genetic resistance to powdery mildew in peas have been reported worldwide, with *er1* being the most widely utilized in breeding programs (Parihar et al., 2022). Although previous studies have identified a suite of defense response genes—including genes encoding chalcone synthases, elicitor-inducible peroxidases, chitinases, phenylalanine ammonia-lyases, defensins, and germin-like proteins—that are upregulated in pea in response to pathogen attack or elicitor treatment (Liu et al., 2003; Curto et al., 2006; Barilli et al., 2014), the downstream molecular mechanisms that underpin pea powdery mildew resistance remain poorly characterized, particularly those governing the coordinated transcriptional and metabolic reprogramming that occurs across the time course of infection. Notably, plant defense is a complex, system-wide process that extends far beyond the activation of isolated resistance genes. Mounting an effective defense requires extensive metabolic reprogramming, in which pathways such as phenylpropanoid biosynthesis play a pivotal role. This pathway produces a diverse array of compounds with multifaceted defense functions. For instance, lignin, flavonoids, and phenolic acids directly contribute to cell wall reinforcement and antimicrobial activity (Naoumkina et al., 2010; Vogt, 2010; Deng and Lu, 2017; Ma and Constabel, 2019; Ortiz and Sansinenea, 2023; Saini et al., 2024).

Modern multi-omics approaches, particularly the integration of transcriptomics and metabolomics, have proven highly effective for deciphering complex plant–pathogen interactions. For example, research on grape resistance to powdery mildew has found that *VqWRKY31* enhanced grapevine powdery mildew resistance by activating the salicylic acid (SA) defense signal and promoting the synthesis of specific disease resistance-related metabolites (e.g., proanthocyanidins, stilbenes, and flavonoids) (Yin et al., 2022). Similarly, in wheat, combined transcriptome and metabolome analyses revealed that resistant varieties exhibit stronger expression of flavonoid biosynthesis genes and higher flavonoid contents upon powdery mildew infection compared with susceptible lines (Xu et al., 2023).

Based on these findings, we hypothesized that resistant and susceptible pea varieties exhibited distinct gene expression and metabolite accumulation patterns in response to powdery mildew infection, particularly within secondary metabolic pathways. To test this hypothesis, a time-course analysis (0, 3, and 6 days post-inoculation) was conducted comparing leaf responses in a susceptible and a resistant pea variety using RNA-seq and non-targeted metabolomics. The objectives of this study were to: (1) identify dynamic changes in gene expression and metabolite profiles during infection; (2) pinpoint key pathways differentiating resistant from susceptible responses; and (3) integrate transcriptomic and metabolomic data to generate a more coherent model of pea defense mechanisms. Our findings provide a foundational molecular resource for understanding pea–powdery mildew interactions and identify potential targets for marker-assisted breeding and the development of sustainable biocontrol strategies.

## 2 Materials and methods

### 2.1 Plant materials and experimental design

The pea varieties Longwan 3 (S3008; susceptible to *E. pisi*) and Longwan 5 (X9002; resistant to *E. pisi*) were used as experimental materials. These two varieties are not isogenic lines and are derived from independent hybrid breeding combinations with distinct genetic backgrounds. The stable high susceptibility of Longwan 3 and high resistance of Longwan 5 to *E. pisi* have been validated by published studies (Lu et al., 2016; Yang et al., 2022b), and the typical stable phenotypic characteristics of the two varieties after inoculation are shown in **Supplementary Figure 1**. The pea seeds were sterilized with 70% (v/v) ethanol for 30 s, followed by surface sterilization with 0.1% (w/v) sodium hypochlorite solution for 5 min, and then rinsed 4–5 times with sterile distilled water to remove residual disinfectant. The sterilized seeds were soaked in autoclaved distilled water for 12–24 h to promote uniform germination, then planted in pots filled with potting soil. All plants were grown in a climate-controlled greenhouse with a 14 h light/10 h dark photoperiod and a constant temperature of 20 ± 2 °C.

Fresh *E. pisi* conidia were harvested from heavily infected leaves of Longwan 3 plants at 7–10 days post-inoculation (dpi, the peak sporulation stage to ensure high conidial viability), and suspended in sterile deionized water containing 0.02% (v/v) Tween 20. The conidial suspension was adjusted to a final concentration of 1 × 10^7^ spores/mL using a hemocytometer for the inoculation of 3-week-old pea plants (Deng et al., 2022). The conidial suspension was uniformly sprayed onto the adaxial surface of all leaves of the resistant (Longwan 5, R) and susceptible (Longwan 3, S) pea varieties. Inoculated plants were first incubated in a growth chamber with 90% relative humidity for 24 h in complete darkness to facilitate synchronous conidial germination and host penetration, then returned to the climate-controlled greenhouse under the original growth conditions. Inoculated plants were sampled at 3 dpi (D3) and 6 dpi (D6), while 0 dpi (D0) samples were collected immediately before inoculation. The three time points (0, 3, and 6 dpi) were selected to represent the pre-infection baseline, the early infection stage, and the established infection stage, respectively, based on the microscopically validated infection kinetics of *E. pisi* on pea (Smith et al., 1996; Fondevilla et al., 2006; Bhosle et al., 2019; Sharma et al., 2019). All collected samples were immediately frozen in liquid nitrogen, and stored at -80 °C until transcriptomic and non-targeted metabolomic analyses. Three biological replicates were included for each group in the transcriptomic analysis, and six biological replicates per group were used for non-targeted metabolomic analysis. Samples for each biological replicate were collected from a minimum of 18 individual plants.

### 2.2 Transcriptomic analysis

Total RNA was extracted from collected samples using the Universal RNA Extraction Kit (R401; Genepioneer Biotechnologies Co. Ltd., Nanjing, China). RNA degradation, contamination, and purity were monitored and checked using agarose gel electrophoresis (1%) and the NanoPhotometer^®^ spectrophotometer (IMPLEN, Munich, Germany). RNA concentration was measured using the Qubit^®^ RNA Assay Kit in a Qubit^®^ 2.0 Fluorometer (Life Technologies, CA, USA). RNA integrity was assessed using the RNA Nano 6000 Assay Kit of the Agilent Bioanalyzer 2100 system (Agilent Technologies, CA, USA). High-quality RNA samples were delivered to Genepioneer Biotechnologies Co. Ltd. (Nanjing, China) for cDNA library construction and sequencing with PE150 using the Illumina NovaSeq 6000 platform (Illumina Inc., San Diego, CA, USA) according to the standard procedure.

Raw sequencing reads were generated by base calling from the raw image files of high-throughput sequencing. Raw reads were cleaned by removing reads containing adapters, poly-N, and low-quality reads using in-house Perl scripts. Concurrently, Q20, Q30, GC content, and sequence duplication levels of the clean data were calculated. All the downstream analyses were based on high-quality clean reads. The reference genome was downloaded from NCBI GenBank, version number GCA_964186695.1_JIC_Psat_v1.3. The index of the reference genome was built and paired-end clean reads were aligned to the reference genome using HISAT2 (v2.1.0). StringTie was used to construct and identify both known and novel transcripts from HISAT2 alignment results. Picard tools (v1.41) and Samtools (v0.1.18) were used to sort, remove duplicated reads, and merge the bam alignment results of each sample. StringTie was used to count the number of reads mapped to each gene.

Principal component analysis (PCA) of samples was conducted using the ‘vegan’ package of R software. The relative expression levels of genes were normalized and expressed as transcripts per kilobase million (TPM) values (**Supplementary Table S1**). The DESeq2 package (v1.42.0) in R was used to identify differentially expressed genes (DEGs) between all nine pairwise comparison groups, based on the raw read counts (Love et al., 2014). Multiple testing correction was performed using the Benjamini-Hochberg false discovery rate (FDR) method (Benjamini and Hochberg, 1995). Genes with an adjusted *P*-value (FDR) < 0.05 and an absolute log2 fold-change value (|log_2_ FC|) ≥ 1 were defined as significant DEGs (Ming et al., 2025; Nath et al., 2025). To eliminate the influence of inherent genetic background differences between the two non-isogenic lines, we applied a two-step filtering strategy. First, pathogen-responsive DEGs were identified via intra-variety temporal comparisons (SD0 vs. SD3, SD0 vs. SD6, RD0 vs. RD3, RD0 vs. RD6), retaining genes with significant expression changes upon *E. pisi* inoculation. Second, resistance-specific DEGs were then identified via inter-variety comparisons at the same time point (SD3 vs. RD3, SD6 vs. RD6), further excluding genes with significant inherent differences at 0 dpi (SD0 vs. RD0). The final DEGs used for downstream functional analysis were those specifically induced by *E. pisi* infection and showing significant differential responses between the resistant and susceptible varieties. Gene Ontology (GO) enrichment analysis of the final filtered DEGs was conducted using the ‘GOseq’ R package based on the Wallenius non-central hyper-geometric distribution (Young et al., 2010). Kyoto Encyclopedia of Genes and Genomes (KEGG) pathway enrichment analysis was performed using KOBAS software (Mao et al., 2005). For supplementary robustness verification of the enrichment results across all comparison groups, we further filtered the DEGs of each group, retaining high-confidence transcripts with a mean TPM ≥ 5. For this independent verification, KEGG Orthology (KO) annotation was performed using the KEGG Automatic Annotation Server (KAAS), and KEGG pathway enrichment analysis was subsequently conducted for all comparison groups using this filtered high-confidence DEG set.

### 2.3 Metabolomic analysis

Frozen leaf samples from each time point and group were used for non-targeted metabolomic analysis. Six biological replicates were included for each group in metabolomic analysis, with each replicate consisting of mixed leaf tissue from a minimum of 18 individual plants. Samples (100 mg) frozen in liquid nitrogen were thoroughly crushed and placed in an Eppendorf (EP) tube, and 500 μL of 80% methanol aqueous solution (ThermoFisher, USA) was added to the EP tube. The mixed samples were vortexed, incubated on ice for 5 min, and then centrifuged at 15,000 ×*g* for 20 min at 4°C. The supernatant was diluted with ddH_2_O (ThermoFisher, USA; mass spectrometry grade) to a methanol content of 53%. The extracts were centrifuged at 15,000 ×*g* and 4°C for 20 min, and then the supernatant was collected for the liquid chromatography-mass spectrometry (LC-MS) analysis by Genepioneer Biotechnologies Co. Ltd. (Nanjing, China).

The extracts were analyzed using an LC-MS system equipped with a Hypesil Gold column (C18, ThermoFisher, USA; 100 × 2.1 mm, 1.9 μm). The column oven was set to 40°C. The flow rate was 0.2 mL/min. The mobile phase consisted of solvent A and solvent B. In positive ion mode, solvent A was 0.1% formic acid (ThermoFisher, USA); in negative ion mode, solvent A was 5 mM ammonium acetate (pH 9.0; ThermoFisher, USA). In both modes, solvent B was methanol (ThermoFisher, USA). The solvent gradient program was performed as follows: 2% B, 1.5 min; 2-85% B, 3.0 min; 85-100% B, 10.0 min; 100-2% B, 10.1 min; 2% B, 12.0 min. MS/MS analysis was performed on a Q Exactive™ HF-X mass spectrometer (ThermoFisher, USA) operated in positive and negative ion modes. The key parameters were set as follows: spray voltage, 3.5 kV; auxiliary gas flow rate, 10 L/min; sheath gas flow rate, 35 psi; S-lens RF level, 60; capillary temperature, 320 °C; auxiliary gas heater temperature, 350 °C; mass scan range, 100–1500 m/z; acquisition mode, full scan with data-dependent MS/MS. The original data were processed using the Compound Discoverer 3.1 (CD 3.1, ThermoFisher) for peak alignment, peak picking, and metabolite identification. Peak alignment was performed with a retention time deviation of 0.2 min and a mass deviation of 5 ppm. Peak picking was performed based on a signal-to-noise ratio of 3, signal strength deviation of 30%, and mass deviation of 5 ppm. Quantification of the peak area and integration of the target ion were then conducted. Then, the molecular formula of each metabolite was predicted based on the molecular ion peaks and MS/MS fragment ions, and metabolite identification was performed by matching against the PubChem Database, Chemical Entities of Biological Interest (ChEBI) Database, NIKKAJI Database, KEGG Database, and Human Metabolome Database (HMDB). All annotated metabolites were classified as Metabolomics Standards Initiative (MSI) Level 2 (putatively annotated compounds, matched to reference MS/MS spectra in public databases), while metabolites with no database matches were classified as MSI Level 4 (unidentified compounds).

PCA and hierarchical clustering analysis (HCA) were performed using the ‘vegan’ package and ‘ComplexHeatmap’ package of R software, respectively (Gu et al., 2016). Orthogonal partial least squares discriminant analysis (OPLS-DA) was conducted based on the variable importance in the projection (VIP) value in the test model using the ‘MetaboAnalystR’ package of R software (Chong and Xia, 2018). PCA, HCA, and OPLS-DA were performed to assess the metabolic differences between sample groups. The robustness of OPLS-DA models was evaluated using R²Y and Q² values, along with permutation testing (200 random permutations). Differentially accumulated metabolites (DAMs) were defined as metabolites with a *P*-value < 0.05 and a VIP score > 1.0. Consistent with the transcriptomic analysis strategy, we adopted the same two-step filtering strategy to eliminate the interference of genetic background: first, identify pathogen-responsive DAMs via intra-variety temporal comparisons; second, screen for resistance-related DAMs via inter-variety comparisons at the same time point, excluding metabolites with inherent differences at 0 dpi. KEGG pathway enrichment analysis of the final filtered DAMs was performed for all nine pairwise comparison groups using the KEGG database. Integrated transcriptomic and metabolomic analysis was performed based on the KEGG pathway annotation. Pearson correlation coefficients between the expression levels of DEGs (TPM) and the relative abundances of DAMs in the phenylpropanoid biosynthesis pathway were calculated using the cor.test function in R, with *P*-values < 0.05 considered statistically significant. Correlation heatmaps were generated using the ‘pheatmap’ package of R software.

## 3 Results

### 3.1 Transcriptional profiling of pea leaves in response to powdery mildew infection

To determine the impact of powdery mildew infection on gene expression in pea, transcriptome analysis was performed on leaves of resistant (R) and susceptible (S) varieties sampled at 0 (D0), 3 (D3), and 6 (D6) days post-inoculation. A total of 119.74 Gb of sequencing data was obtained by transcriptome sequencing. After removing the low-quality reads, the clean data for each sample reached 6.05 Gb or above, with 20.34–25.34 million clean read pairs per sample (derived from 20.46–25.34 million raw read pairs) (**Supplementary Table S2**). The Q30 percentage and GC content were 94.70%–95.41% and 42.78%–43.64% (**Supplementary Table S2**), respectively, indicating relatively high quality of the transcriptome sequencing data. The clean reads were mapped to the reference genome with a mapping rate of 92.01%–94.22% (**Supplementary Table S2**), and a total of 34566 genes were functionally annotated in the databases.

**Figure 1:**
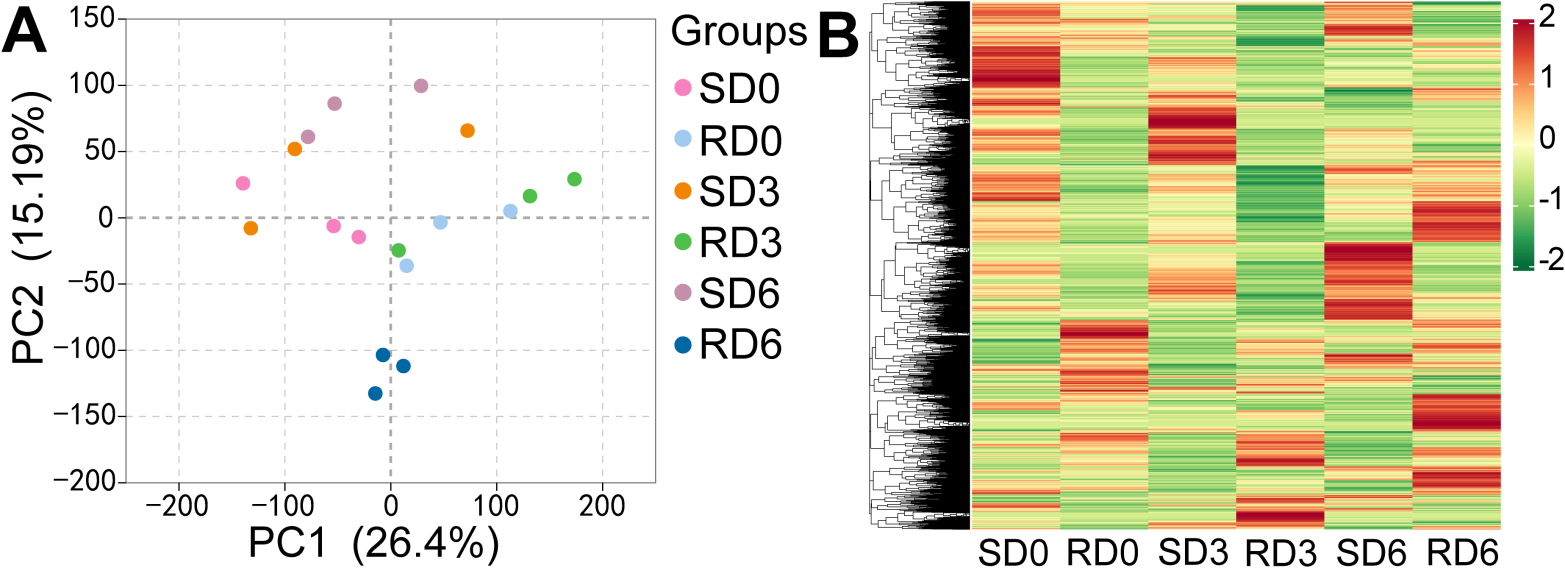
Transcriptional profiles of pea leaves from powdery mildew-susceptible (Longwan 3) and -resistant (Longwan 5) varieties at different days post-inoculation (dpi) (*n* = 3). **(A)** Principal component analysis (PCA) of transcriptome data. **(B)** Hierarchical clustering heatmap of genome-wide gene expression profiles. SD0, SD3, and SD6, susceptible variety (Longwan 3) at 0, 3, and 6 dpi, respectively. RD0, RD3, and RD6, resistant variety (Longwan 5) at 0, 3, and 6 dpi, respectively.

The correlation heatmap analysis revealed high concordance among biological replicates for each sample group, indicating high reproducibility of the transcriptome data (**Supplementary Figure S2**). Principal component analysis (PCA) was performed to assess the transcriptional variation across all samples. The first principal component (PC1) explained 26.4% of the total variance, and the second principal component (PC2) explained 15.19% of the variance, with clear separation between resistant and susceptible varieties along PC2 (**Figure 1A**). Permutational multivariate analysis of variance (PERMANOVA) revealed that inoculation treatment (*R*^2^ = 57.524%, *P* = 0.001) and pea variety (*R*^2^ = 22.931%, *P* = 0.004) both had significant effects on the leaf transcriptional profile. Hierarchical clustering analysis (HCA) also showed that there were differences in the transcriptional profiles of leaves under different treatments (**Figure 1B**). Moreover, there was no significant change in the transcriptional profile of the leaves at different sampling stages (*R*^2^ = 15.193%, *P* = 0.211).

**Figure 2:**
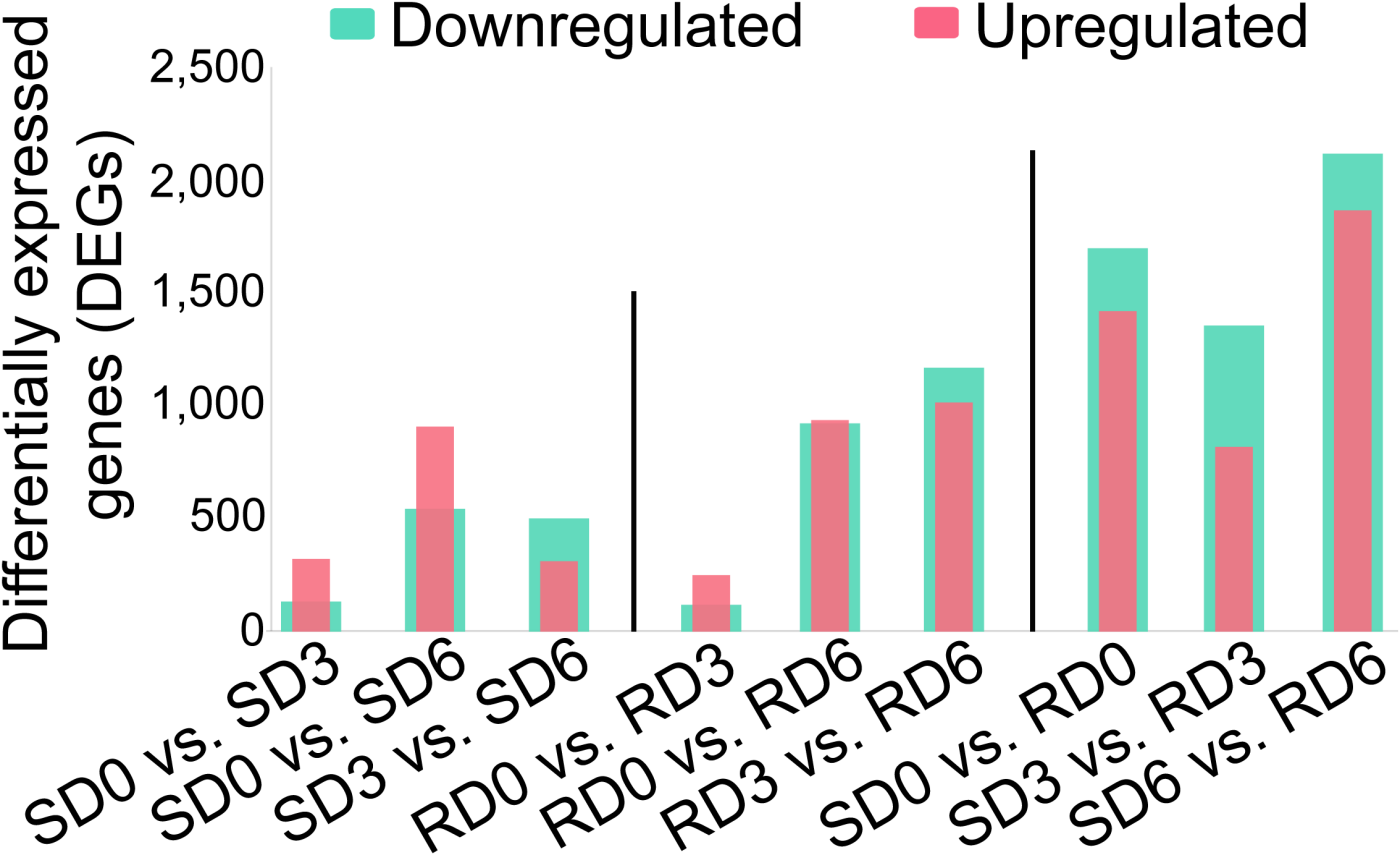
Number of upregulated and downregulated differentially expressed genes (DEGs) in pea leaves across nine pairwise comparisons. DEGs were defined as genes with false discovery rate (FDR) < 0.05 and |log2 fold change (|log2FC|) ≥ 1. SD0, SD3, and SD6, susceptible variety (Longwan 3) at 0, 3, and 6 days post-inoculation (dpi), respectively. RD0, RD3, and RD6, resistant variety (Longwan 5) at 0, 3, and 6 dpi, respectively.

Using |Log_2_FC| ≥ 1 and FDR < 0.05 as the criteria for screening differentially expressed genes (DEGs), the transcriptional responses of each variety to *E. pisi* infection via intra-variety temporal comparisons were first analyzed (**Figure 2**). In the susceptible variety, a total of 455 (SD0 vs. SD3), 1453 (SD0 vs. SD6), and 814 (SD3 vs. SD6) DEGs were identified, with the number of pathogen-responsive DEGs peaking at 6 dpi, indicating a delayed defense response. In the resistant variety, the number of pathogen-responsive DEGs increased gradually with infection progression, with 369 (RD0 vs. RD3), 1861 (RD0 vs. RD6), and 2186 (RD3 vs. RD6) DEGs identified respectively, reflecting a sustained and stepwise activation of defense responses in the resistant variety.

Subsequently, we performed inter-variety comparisons at the same time point to characterize the genotypic differences between the two varieties. For the full pairwise comparisons between the resistant and susceptible varieties (SD0 vs. RD0, SD3 vs. RD3, and SD6 vs. RD6), the total number of DEGs were 3121, 2176, and 3987, respectively, including 1421, 819, and 1868 upregulated genes and 1700, 1357, and 2119 downregulated genes. To eliminate the interference of inherent genetic background differences, we excluded the 3121 DEGs identified in SD0 vs. RD0 (pre-inoculation inherent differences) from the inter-variety comparison results and finally obtained 836 core resistance-related DEGs for SD3 vs. RD3, and 2818 core resistance-related DEGs for SD6 vs. RD6 (**Supplementary Figure S3**).

### 3.2 Functional enrichment analysis of differentially expressed genes

GO functional classification was performed for all DEGs, which were assigned to three main GO categories: cellular component, molecular function, and biological process (**Supplementary Figures S4–S6**). In nine pairwise comparison groups (SD0 vs. SD3, SD0 vs. SD6, SD3 vs. SD6, RD0 vs. RD3, RD0 vs. RD6, RD3 vs. RD6, SD0 vs. RD0, SD3 vs. RD3, and SD6 vs. RD6), the distribution of DEGs was similar. Specifically, in the cellular component category, DEGs were predominantly enriched in the GO terms cell, cell part, and organelle. In molecular function category, DEGs were enriched in catalytic activity and binding terms. For biological process, DEGs were mainly mapped to cellular process, metabolic process, biological regulation, and response to stimulus terms.

**Figure 3:**
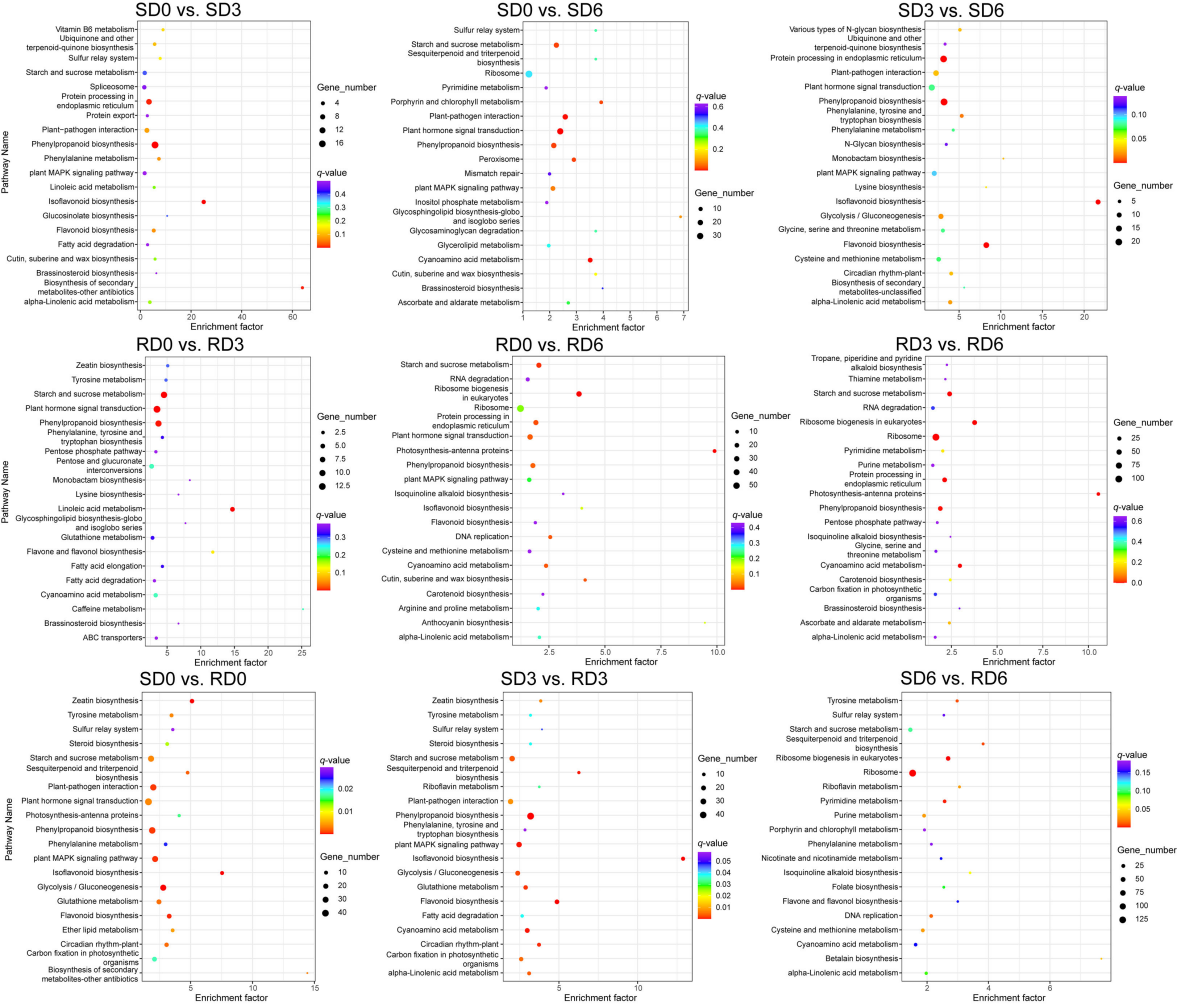
Bubble plot of KEGG pathway enrichment analysis of differentially expressed genes (DEGs) in pea leaves across nine pairwise comparisons (*n* = 3). Pathways with a *P* < 0.05 were considered significantly enriched. SD0, SD3, and SD6, susceptible variety (Longwan 3) at 0, 3, and 6 days post-inoculation (dpi), respectively. RD0, RD3, and RD6, resistant variety (Longwan 5) at 0, 3, and 6 dpi, respectively.

For the KEGG annotation results, DEGs were assigned to six main categories, including cellular processes, environmental information processing, genetic information processing, human diseases, metabolism, and organismal systems (**Supplementary Figure S7**). Among these, the metabolism category accounted for the highest proportion of DEGs, which contained a total of 10 enriched metabolic pathways (**Supplementary Figure S7**). KEGG pathway enrichment analysis of DEGs identified the top 20 significantly enriched pathways (**Figure 3**). In SD0 vs. SD3, SD0 vs. SD6, and SD3 vs. SD6, DEGs were significantly enriched in phenylpropanoid biosynthesis. In RD0 vs. RD3, RD0 vs. RD6, and RD3 vs. RD6, DEGs were significantly enriched in starch and sucrose metabolism and phenylpropanoid biosynthesis. In SD0 vs. RD0 and SD3 vs. RD3, DEGs were significantly enriched in the isoflavonoid biosynthesis, flavonoid biosynthesis, plant MAPK signaling pathway, and phenylpropanoid biosynthesis. In SD6 vs. RD6, DEGs were significantly enriched in the ribosome biogenesis in eukaryotes, ribosome, pyrimidine metabolism, sesquiterpenoid and triterpenoid biosynthesis, and tyrosine metabolism. To verify the robustness of our core conclusion and eliminate the interference of both low-abundance transcriptional noise and inherent genetic background differences between the two varieties, we first retained only high-reliability transcripts with a mean TPM ≥ 5 to exclude low-abundance noise, then we further locked the DEGs specifically responsive to *E. pisi* inoculation via intra-variety temporal comparisons, and excluded all DEGs with significant inherent genetic differences between the two varieties at 0 dpi pre-inoculation. Enrichment analysis of this filtered core DEG set confirmed that the enrichment of our core regulatory pathway, phenylpropanoid biosynthesis, was robust and not driven by low-abundance noise or inherent genetic background differences (**Supplementary Figures S8–S10**).

### 3.3 Metabolomic profiling of pea leaves in response to powdery mildew infection

**Figure 4:**
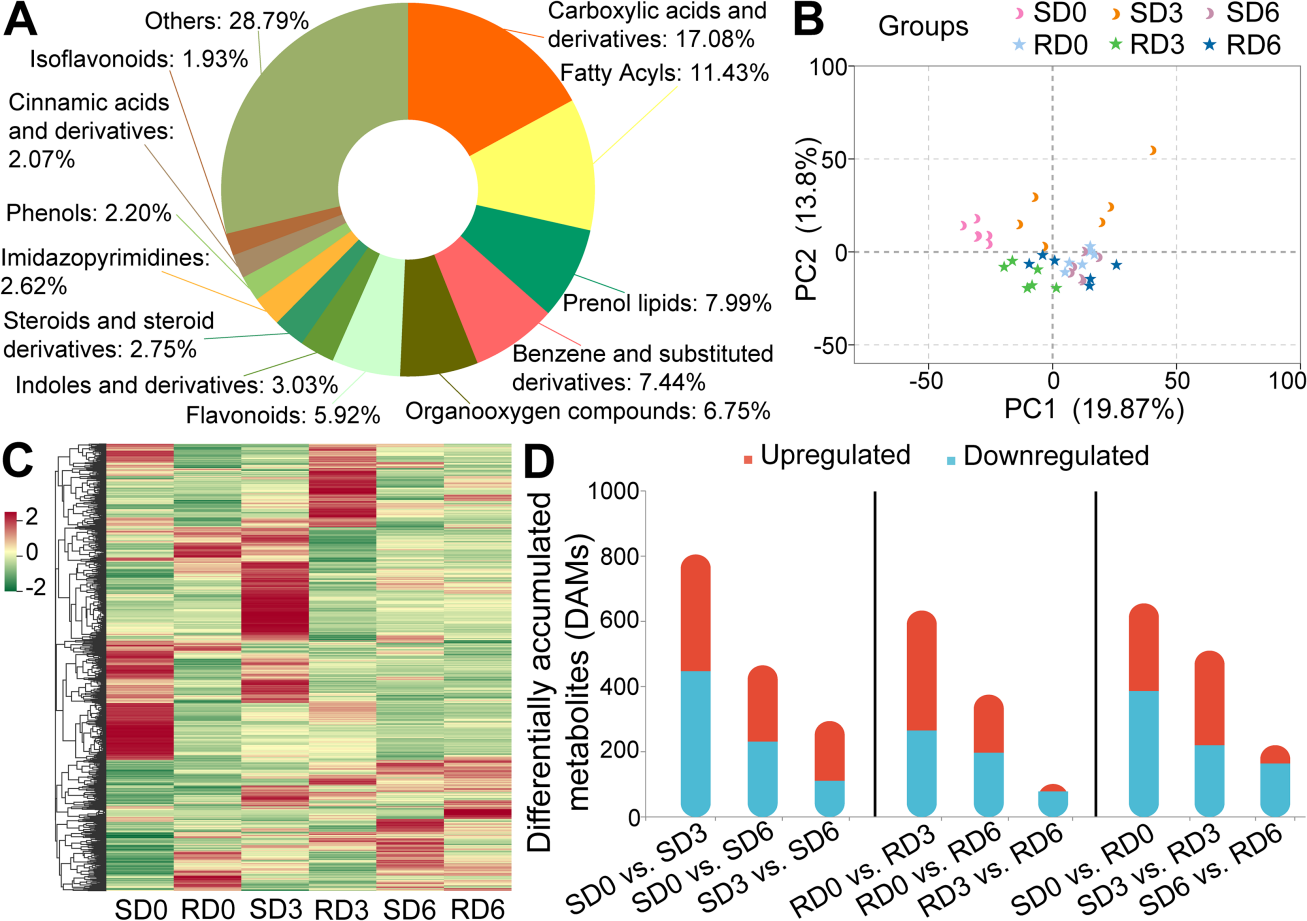
Metabolomic profiles of pea leaves from powdery mildew-susceptible (Longwan 3) and -resistant (Longwan 5) varieties at different stages (*n* = 6). **(A)** Classification of annotated metabolites based on the Human Metabolome Database (HMDB) tertiary classification. **(B)** Principal component analysis (PCA) of metabolomic data. **(C)** Hierarchical clustering heatmap of metabolite accumulation profiles. **(D)** Number of upregulated and downregulated differentially accumulated metabolites (DAMs) across nine pairwise comparisons. SD0, SD3, and SD6, susceptible variety (Longwan 3) at 0, 3, and 6 days post-inoculation (dpi), respectively. RD0, RD3, and RD6, resistant variety (Longwan 5) at 0, 3, and 6 dpi, respectively.

To determine the influence of powdery mildew infection on the metabolite profile of resistant and susceptible pea varieties, non-targeted metabolomic profiling was conducted on pea leaves treated in the same manner as transcriptome sequencing. A total of 1754 metabolites were detected in all samples (**Supplementary Table S3**), and 762 metabolites were clearly annotated and classified in the HMDB database. Based on the tertiary classification from the HMDB database, the annotated metabolites were divided into 12 major categories: carboxylic acids and derivatives (17.08%), fatty acyls (11.43%), prenol lipids (7.99%), benzene and substituted derivatives (7.44%), organooxygen compounds (6.75%), flavonoids (5.92%), indoles and derivatives (3.03%), steroids and steroid derivatives (2.75%), imidazopyrimidines (2.62%), phenols (2.20%), cinnamic acids and derivatives (2.07%), and isoflavonoids (1.93%, **Figure 4A**). The correlation heatmap showed a high correlation between the biological replicates of the samples, indicating high reproducibility among biological replicates within each group (**Supplementary Figure S11**). PCA showed that PC1 and PC2 accounted for 19.87% and 13.8% of the total variables, respectively (**Figure 4B**). PERMANOVA revealed that there were significant differences in the metabolic profiles of the leaves between different treatments (*R*^2^ = 42.637%, *P* = 0.001). In addition, significant metabolic profile differences were observed between leaves of resistant and susceptible pea varieties (*R*^2^ = 6.065%, *P* = 0.037) and at different stages (*R*^2^ = 13.746%, *P* = 0.006). HCA also indicated that there were differences in the accumulation patterns of metabolites in the leaves of resistant and susceptible pea varieties after inoculation with powdery mildew at different stages (**Figure 4C**). To validate the robustness of the OPLS-DA models used for differential metabolite identification, we assessed model performance metrics and performed permutation testing (**Supplementary Table S4**). All models exhibited high R²Y values (ranging from 0.986 to 0.998) and Q² values (ranging from 0.497 to 0.959), indicating good explanatory power and predictive ability. Permutation tests with 200 random permutations confirmed that all models were statistically significant (*P* < 0.05), with no evidence of overfitting.

Using *P* < 0.05 and VIP > 1.0 as the criteria for screening differentially accumulated metabolites (DAMs), pathogen-responsive DAMs were first identified via intra-variety temporal comparisons (**Figure 4D**). In the susceptible variety, 805 (SD0 vs. SD3), 465 (SD0 vs. SD6), and 294 (SD3 vs. SD6) DAMs were identified. In the resistant variety, 633 (RD0 vs. RD3), 375 (RD0 vs. RD6), and 101 (RD3 vs. RD6) DAMs were identified. The number of pathogen-responsive DAMs gradually decreased across time points in both varieties, suggesting a gradual stabilization of metabolic responses after the initial pathogen perturbation.

Subsequently, we performed inter-variety comparisons at the same time point to characterize genotypic metabolic differences. For the full pairwise comparisons between the two varieties (SD0 vs. RD0, SD3 vs. RD3, and SD6 vs. RD6), the total number of DAMs were 655, 510, and 220, respectively, including 269, 290, and 56 upregulated metabolites and 386, 220, and 164 downregulated metabolites (**Figure 4D**). Consistent with the transcriptomic analysis strategy, we excluded the 655 inherent differential metabolites identified in SD0 vs. RD0 and finally obtained 256 core resistance-related DAMs for SD3 vs. RD3, and 78 core resistance-related DAMs for SD6 vs. RD6 (**Supplementary Figure S12**).

### 3.4 KEGG pathway enrichment analysis of differentially accumulated metabolites

**Figure 5:**
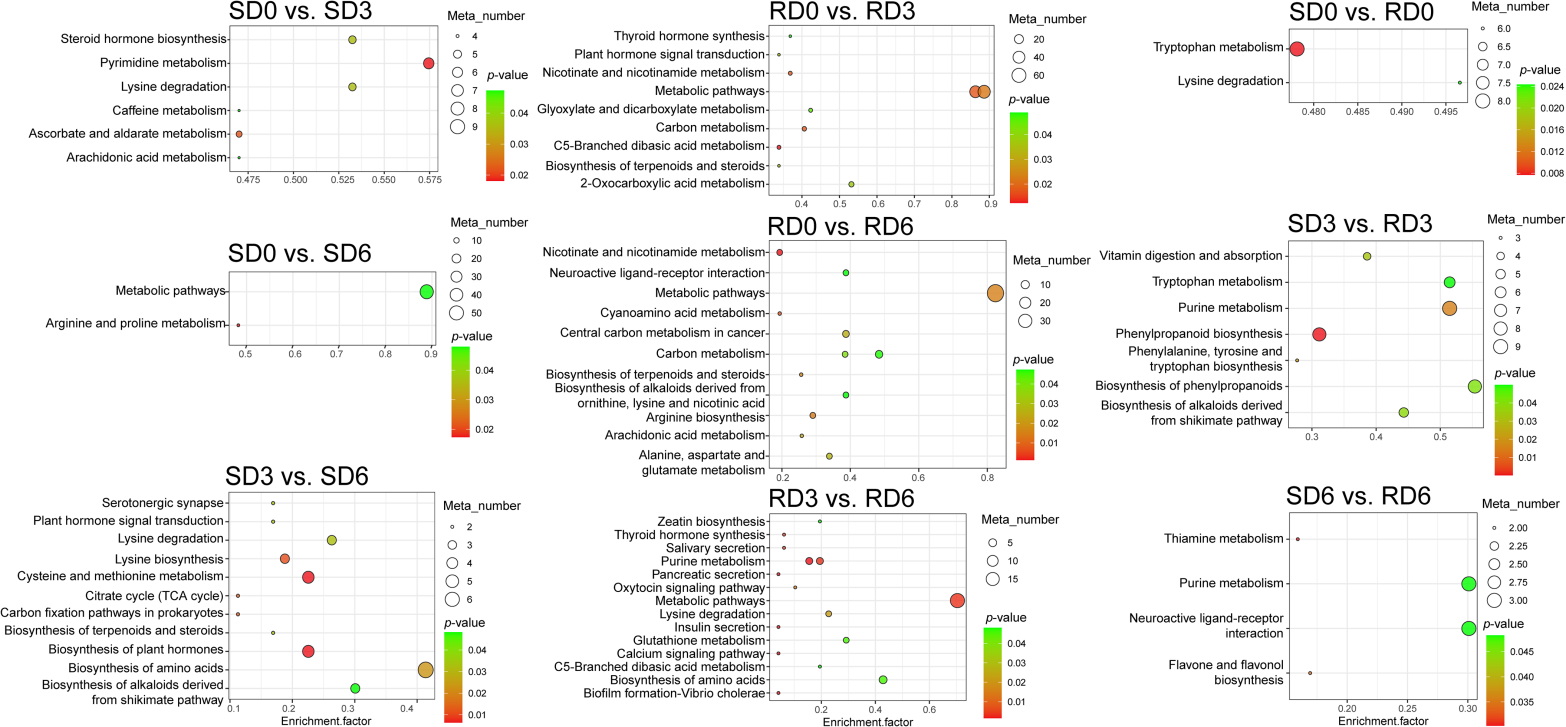
Bubble plot of KEGG pathway enrichment analysis of differentially accumulated metabolites (DAMs) in pea leaves across nine pairwise comparisons (*n* = 6). SD0, SD3, and SD6, susceptible variety (Longwan 3) at 0, 3, and 6 days post-inoculation (dpi), respectively. RD0, RD3, and RD6, resistant variety (Longwan 5) at 0, 3, and 6 dpi, respectively.

KEGG pathway enrichment analysis of all DAMs identified the top 8 significantly enriched pathways (**Figure 5**). In the susceptible varieties, DAMs were significantly enriched in the pyrimidine metabolism in SD0 vs. SD3, metabolic pathways in SD0 vs. SD6, cysteine and methionine metabolism, biosynthesis of plant hormones, and biosynthesis of amino acids in SD3 vs. SD6. In the resistant varieties, DAMs were significantly enriched in the metabolic pathways in RD0 vs. RD3, RD0 vs. RD6, and RD3 vs. RD6. In SD0 vs. RD0, DAMs were significantly enriched in tryptophan metabolism. In SD3 vs. RD3, DAMs were significantly enriched in the biosynthesis of phenylpropanoids, phenylpropanoid biosynthesis, and purine metabolism. In SD6 vs. RD6, DAMs were significantly enriched in the neuroactive ligand-receptor interaction and purine metabolism. To eliminate the interference of inherent genetic background differences, we performed KEGG enrichment analysis on the two-step filtered core resistance-related DAMs. The results showed that the phenylpropanoid biosynthesis pathway was stably detected and ranked among the top differential pathways in both SD3 vs. RD3 and SD6 vs. RD6 after filtering (**Supplementary Figure S13**).

### 3.5 Integrated transcriptomic and metabolomic analysis of phenylpropanoid biosynthesis pathway

**Figure 6:**
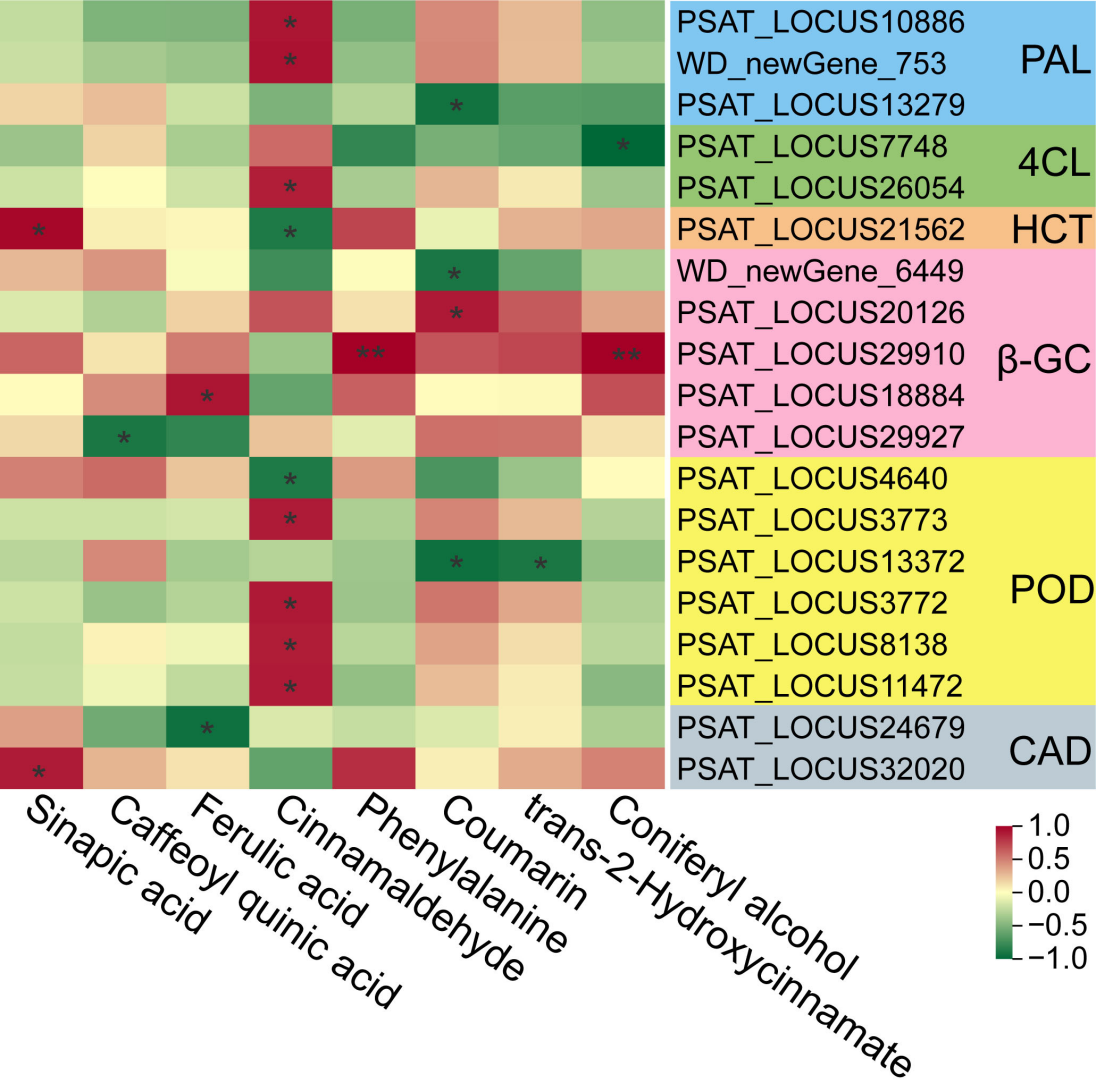
Correlation analysis between key differentially expressed genes (DEGs) and differentially accumulated metabolites (DAMs) in the phenylpropanoid pathway. The color scale represents the Pearson correlation coefficient (r) based on the expression levels of genes (transcripts per kilobase million, TPM) and the relative abundance of metabolites across all samples. Asterisks indicate statistically significant correlations (^*^P < 0.05, ^**^P < 0.01). PAL, phenylalanine ammonia-lyase; 4CL, 4-coumarate-CoA ligase; HCT, shikimate *O*-hydroxycinnamoyltransferase; β-GC, beta-glucosidase; POD, peroxidase; CAD, cinnamyl-alcohol dehydrogenase.

To identify the core regulatory axis driving differential powdery mildew resistance between the two varieties, we performed integrated analysis of DEGs and DAMs, focusing on the consistently enriched phenylpropanoid biosynthesis pathway across multiple comparisons. Pearson correlation analysis was conducted to quantify the coupling between the expression of core structural genes in the pathway and the accumulation of associated defensive metabolites. Pearson correlation analysis revealed significant quantitative coupling between the expression of core phenylpropanoid structural genes and the accumulation of defense-related metabolites (**Figure 6**; **Supplementary Table S5**). Specifically, genes encoding key enzymes, including PAL, 4CL, and POD, exhibited strong positive correlations (r > 0.8, *P* < 0.05) with cinnamaldehyde. Furthermore, specific genes (e.g., certain HCT and CAD transcripts) were positively correlated with sinapic acid, while multiple β-GC-encoding genes showed significant positive associations with key compounds such as ferulic acid, coumarin, phenylalanine, and coniferyl alcohol (r > 0.8, *P* < 0.05 or *P* < 0.01). We also observed negative correlations between specific genes (e.g., certain PAL, 4CL, and POD transcripts) and metabolites like coumarin and cinnamaldehyde, reflecting the complex, multi-branched regulatory network and potential negative feedback loops within the pathway. Overall, these quantitative correlations validate that transcriptional activation of core genes is tightly coupled to metabolite accumulation, providing direct evidence of functional pathway activation during pea resistance.

**Figure 7:**
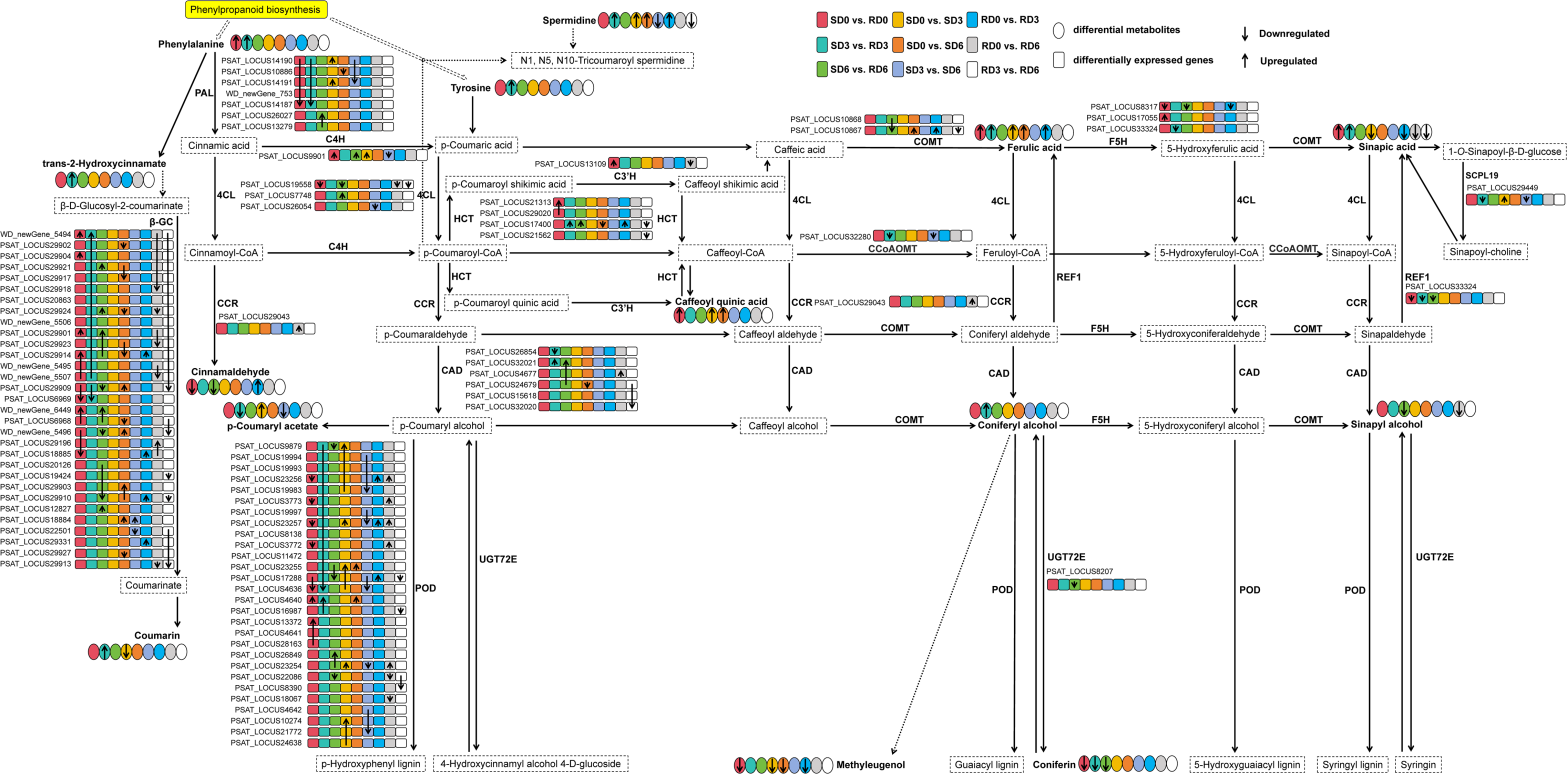
Integrated visualization of differentially expressed genes (DEGs) and differentially accumulated metabolites (DAMs) involved in phenylpropanoid biosynthesis pathway in pea leaves across all pairwise comparisons. Dotted boxes indicate metabolites not detected in this study. Elliptical boxes with different colors represent that the metabolites were detected in each pairwise comparison. Different colored boxes represent DEGs identified in each pairwise comparison. The color of each box corresponds to the pairwise comparison as follows: red represents SD0 vs. RD0, cyan represents SD3 vs. RD3, green represents SD6 vs. RD6, yellow represents SD0 vs. SD3, orange represents SD0 vs. SD6, lilac represents SD3 vs. SD6, blue represents RD0 vs. RD3, light gray represents RD0 vs. RD6, white represents RD3 vs. RD6. The small upward arrow and small downward arrow of the elliptical boxes (metabolites) and boxes (genes) indicate the upregulation and downregulation of DEGs and DAMs, respectively. For all pairwise comparisons, the direction of regulation (up/down) is relative to the latter group listed in the comparison name. Solid arrows, single biosynthetic steps. Dotted arrows, multiple steps. SD0, SD3, and SD6, susceptible variety (Longwan 3) at 0, 3, and 6 days post-inoculation (dpi), respectively. RD0, RD3, and RD6, resistant variety (Longwan 5) at 0, 3, and 6 dpi, respectively. PAL, phenylalanine ammonia-lyase; 4CL, 4-coumarate-CoA ligase; C4H, cinnamate 4-hydroxylase; REF1, coniferyl-aldehyde dehydrogenase; HCT, shikimate *O*-hydroxycinnamoyltransferase; C3*’*H, 5-*O*-(4-coumaroyl)-D-quinate 3*’*-monooxygenase; COMT, caffeic acid 3-*O*-methyltransferase; CAD, cinnamyl-alcohol dehydrogenase; POD, peroxidase; UGT72E, coniferyl-alcohol glucosyltransferase; β-GC, beta-glucosidase; F5H, ferulate-5-hydroxylase; CCoAOMT, caffeoyl-CoA *O*-methyltransferase; SCPL19, serine carboxypeptidase-like 19; CCR, cinnamoyl-CoA reductase.

The significantly enriched DAMs were involved in multiple metabolic pathways across most pairwise comparisons. Given the consistent enrichment of the phenylpropanoid biosynthesis pathway in both transcriptomic and metabolomic datasets across multiple comparisons, we mapped all 90 DEGs and 14 DAMs involved in this pathway to the canonical metabolic pathway (**Figure 7**). Specifically, in SD0 vs. RD0, the expression of genes encoding trans-cinnamate 4-monooxygenase (C4H), hydroxycinnamoyl transferase (HCT), 3′-hydroxylase (C3′H) was significantly upregulated, accompanied by increased accumulation of L-phenylalanine, sinapic acid, chlorogenic acid, and ferulic acid. Conversely, genes encoding PAL, 4-coumarate-CoA ligase (4CL), coniferyl-aldehyde dehydrogenase (REF1) were downregulated, along with decreased levels of coniferin, methyl eugenol, and cinnamaldehyde. Genes encoding β-glucosidase (β-GC), ferulate 5-hydroxylase (F5H), and peroxidase (POD) exhibited mixed expression patterns (both upregulation and downregulation). In SD3 vs. RD3, genes encoding HCT were upregulated alongside increased levels of sinapic acid, spermidine, ferulic acid, coumarin, trans-2-hydroxycinnamate, coniferyl alcohol, L-tyrosine, and L-phenylalanine. Meanwhile, genes encoding PAL, F5H, REF1, caffeoyl-CoA *O*-methyltransferase (CCoAOMT), and serine carboxypeptidase-like 19 (SCPL19) were downregulated, consistent with decreased coniferin and p-coumaryl acetate contents. Genes encoding β-GC, cinnamyl alcohol dehydrogenase (CAD), and POD showed mixed expression patterns. In SD6 vs. RD6, genes encoding caffeate/5-hydroxyferulate *O*-methyltransferase (COMT), F5H, REF1, coniferyl-alcohol glucosyltransferase (UGT72E) were downregulated, as were the metabolites coniferin, sinapyl alcohol, and cinnamaldehyde. In contrast, genes encoding PAL, C4H, HCT, and CAD were upregulated. Genes encoding β-GC, 4CL, and POD showed mixed expression patterns.

In SD0 vs. SD3, the expression of genes encoding PAL, C4H, SCPL19, POD was significantly upregulated, concurrent with the accumulation of p-coumaryl acetate, chlorogenic acid, ferulic acid, and spermidine, whereas levels of sinapic acid, methyl eugenol, and coumarin decreased. In SD0 vs. SD6, genes encoding POD and COMT were upregulated alongside chlorogenic acid, spermidine, and ferulic acid accumulation, while genes encoding PAL, HCT, CAD were downregulated, matching the decrease in methyl eugenol. In SD3 vs. SD6, genes encoding POD, CCoAOMT, SCPL19, 4CL, C4H, PAL were significantly downregulated, as were p-coumaryl acetate and spermidine contents. Genes encoding β-GC showed mixed expression patterns in both SD0 vs. SD6 and SD3 vs. SD6 comparisons.

In RD0 vs. RD3, genes encoding β-GC, HCT, COMT, POD were upregulated, accompanied by increased spermidine, ferulic acid, and cinnamaldehyde. Conversely, genes encoding F5H were downregulated, along with reduced sinapic acid and methyl eugenol levels. In RD0 vs. RD6, genes encoding 4CL and C3′H were downregulated together with sinapic acid and sinapyl alcohol, while genes for CAD were upregulated. Genes encoding β-GC and POD showed mixed expression patterns. In RD3 vs. RD6, genes encoding 4CL, HCT, COMT, CAD, POD, β-GC were downregulated, consistent with decreased levels of spermidine and sinapic acid.

Collectively, these results demonstrate that the transcriptional reprogramming of key phenylpropanoid biosynthetic genes directly drives the accumulation of defensive metabolites, which underlies the differential powdery mildew resistance between the two pea varieties. For instance, in some pairwise comparison groups, the upregulation of synthesis-related genes (such as *C4H*) coincided with increased relative contents of ferulic acid. However, discrepancies between gene expression and metabolite content were also observed. For instance, genes encoding β-GC, POD, and other enzymes showed mixed regulation across multiple comparisons, yet the trends of related metabolites did not always align perfectly, suggesting the presence of complex post-transcriptional, post-translational, and metabolic feedback regulation mechanisms that modulate the relationship between gene transcription and metabolite accumulation in this pathway.

## 4 Discussion

In this study, we employed an integrated transcriptomic and metabolomic approach to investigate the dynamic molecular and metabolic responses of powdery mildew-susceptible and -resistant pea varieties to pathogen infection. Our comprehensive dataset revealed a central and differentially regulated role for phenylpropanoid biosynthesis in differentiating resistant and susceptible interactions. Transcriptome analysis showed that the number of DEGs between resistant and susceptible varieties (SD0 vs. RD0, SD3 vs. RD3, and SD6 vs. RD6) fluctuated across the infection time course (**Figure 2**), indicating that distinct gene sets were involved in different stages of the defense response. The gradual increase in DEGs in the resistant variety over time (**Figure 2**) reflects a sustained, stepwise activation of defense responses, consistent with previous findings in pea (Barilli et al., 2014). In contrast, the fluctuating DEG pattern in the susceptible variety (**Figure 2**) suggests a disorganized, ineffective initial defense that fails to be sustained over the course of infection. Collectively, these results indicate that the resistant variety maintains a more stable and persistent transcriptional reprogramming that is essential for mounting effective powdery mildew resistance. Consistent with the transcriptome results, the number of DAMs between resistant and susceptible varieties decreased over the infection time course (**Figure 4D**), possibly indicating a convergence of metabolic profiles driven by pathogen manipulation in later infection stages, especially in susceptible plants (Ullah et al., 2020). Within both varieties, the number of DAMs progressively decreased across time points (**Figure 4D**), suggesting a gradual stabilization of metabolic responses after the initial pathogen perturbation, and a shift towards more targeted, specialized defense mechanisms (Fondevilla et al., 2011; Patel et al., 2020).

KEGG pathway enrichment analysis consistently highlighted that DEGs and DAMs were predominantly enriched in the phenylpropanoid biosynthesis pathway across multiple comparisons (**Figure 3** and **5**), underscoring its central role in pea defense against powdery mildew. This pathway produces an array of compounds with multifaceted defense functions. For instance, lignin, flavonoids, and phenolic acids contribute to cell wall reinforcement and direct antimicrobial activity (Naoumkina et al., 2010; Vogt, 2010; Deng and Lu, 2017; Ma and Constabel, 2019; Ortiz and Sansinenea, 2023; Saini et al., 2024), jointly enhancing host defense against pathogen invasion. The critical role of phenylpropanoids and flavonoids in powdery mildew resistance has been well-demonstrated in other crops, such as grape and wheat (Yin et al., 2022; Xu et al., 2023). Notably, phenylpropanoid-derived phenolics are well-documented as key components of root exudates that modulate rhizosphere microbial communities (Fang et al., 2013; Zhou et al., 2018; Xia et al., 2021; An et al., 2022). While our study focused exclusively on foliar tissue responses to powdery mildew, we hypothesize that the systemic induction of phenylpropanoid biosynthesis observed here may also modulate belowground biotic interactions. However, this hypothesis requires dedicated experimental validation in future work.

Within the phenylpropanoid biosynthesis pathway, the transcript levels of key enzyme-encoding genes and the accumulation of associated defense metabolites showed distinct patterns between resistant and susceptible varieties. The expression of the *PAL* gene showed dynamic regulation, being initially suppressed in the resistant variety but strongly induced at later infection stages (**Figure 7**), suggesting its time-dependent role in the defense response. Specifically, PAL may act as a molecular switch that redirects metabolic flux between different branches of the phenylpropanoid pathway at different stages of pathogen infection. Conversely, *C4H* transcripts showed upregulation in the resistant variety at both early (SD0 vs. RD0) and late (SD6 vs. RD6) stages of infection (**Figure 7**), which likely drives the sustained accumulation of defensive metabolites in the resistant variety. Our results are consistent with previous research on biological control, such as the combined application of *Ascophyllum nodosum* extract and chitosan, which significantly reduced the severity of pea powdery mildew by upregulating *PAL* and *C4H* expression, and activating SA and jasmonic acid (JA) signaling pathways (Patel et al., 2020). These findings from our study indicate that the phenylpropanoid pathway not only functions as a direct defense mechanism, but also interacts with hormone signaling networks to enhance systemic acquired resistance in pea. In addition, increased accumulation of ferulic acid and sinapic acid in resistant varieties in the early stages of infection (SD0 vs. RD0 and SD3 vs. RD3; **Figure 7**) suggests that resistant pea plants fortify their cell walls through enhanced lignification, and accumulate antimicrobial compounds to impede pathogen invasion and spread (Kusumoto, 2005; Rashad et al., 2020; Ninkuu et al., 2022).

Through integrative pathway analysis, we observed that changes in gene expression generally corresponded with the accumulation of their respective metabolites, particularly for early biosynthetic genes and their direct products. For example, the upregulated expression of *C4H* correlated with increased levels of ferulic acid in multiple pairwise comparisons (**Figure 7**). However, the relationship between gene transcription and metabolite accumulation was not always linear. For instance, genes encoding β-GC and POD showed mixed expression patterns (both upregulation and downregulation of different gene family members) in multiple comparisons, with no consistent correlation with the levels of related metabolites (**Figure 7**). This isoform-specific expression pattern suggests that different family members may play distinct, even opposing, roles in the defense response, with functional specialization across different infection stages. The inconsistent expression patterns of these genes and their metabolites may reflect the dynamics of plant defense responses, where multiple regulatory mechanisms are activated at different infection stages, including post-transcriptional regulation, post-translational modification, enzyme activity modulation, substrate channeling, and feedback inhibition (Hemmerlin, 2013; Zhang et al., 2022; Pandey and Gayen, 2024). This emphasizes the variety-specific and time-dependent mechanism of pea resistance to powdery mildew, and suggests that resistant varieties mount a more effective, coordinated metabolic reprogramming in response to pathogen infection. Indeed, plant defense responses involve intricate signaling and regulatory networks that coordinate the precise production of defensive compounds (Rojo et al., 2003; Maag et al., 2015; Kaur et al., 2022; Upadhyay et al., 2025). While phenylpropanoid biosynthesis was the predominant pathway identified in this study, the enrichment of other pathways provides a more holistic view of the pea defense response. The enrichment of the MAPK signaling pathway (e.g., SD3 vs. RD3; **Figure 3**) is consistent with its known role in transducing pathogen recognition signals and activating downstream defense responses in plants (Meng and Zhang, 2013). Changes in purine metabolism (SD3 vs. RD3 and SD6 vs. RD6) and pyrimidine metabolism (SD0 vs. SD3; **Figure 5**) likely reflect shifts in energy metabolism and nucleotide pools required to support rapid transcriptional reprogramming and defense signaling during infection. The enrichment of cysteine and methionine metabolism and biosynthesis of amino acids in the susceptible variety (SD3 vs. SD6; **Figure 5**) could suggest either a pathogen-driven manipulation of host nutrition to support pathogen growth, or a non-specific stress-induced metabolic shift. The enrichment of plant hormone biosynthesis in the susceptible variety (SD3 vs. SD6; **Figure 5**) points to a critical role for hormonal signaling networks, such as SA and JA, in determining the outcome of pea–powdery mildew interactions.

This study provides valuable molecular resources and biomarkers for breeding pea cultivars with enhanced powdery mildew resistance. The identification of key DEGs (e.g., genes encoding PAL, C4H, and HCT) and DAMs (e.g., ferulic acid and sinapic acid) associated with the phenylpropanoid pathway offers promising candidate targets for marker-assisted selection or gene editing for disease resistance. Furthermore, the key defense metabolites identified in this study could be explored as natural priming agents or elicitors to induce plant innate immunity, or serve as biochemical markers for screening resistant germplasm. These findings open new avenues for developing novel green control strategies based on plant immune priming, thereby reducing agricultural reliance on synthetic chemical fungicides. While this study provides transcriptomic and metabolomic insights into pea–powdery mildew interactions, certain limitations must be acknowledged. First, our experimental design did not include a mock-inoculated control group. While we used 0 dpi samples as the experimental baseline to eliminate inherent transcriptomic and metabolic differences between the two pea varieties at the pre-inoculation stage, this design cannot fully disentangle pathogen-specific defense responses from general stress responses triggered by the mechanical inoculation procedure and environmental manipulation. Thus, we acknowledge that a subset of the early molecular changes observed in this study may reflect universal stress responses rather than pathogen-specific defense mechanisms, which limits our ability to resolve infection-specific regulatory events in the earliest stages of pathogen challenge. Future studies should incorporate a parallel mock-inoculated control group to more precisely dissect the pathogen-specific molecular and metabolic reprogramming events that drive pea powdery mildew resistance. Second, our experiments were conducted under controlled greenhouse conditions, which are essential for mechanistic clarity, but the findings require validation under field conditions, where environmental variables and the soil microbiome can strongly modulate plant immunity. Third, our study focused exclusively on leaf tissue, the primary site of powdery mildew infection, but the findings should be extended to pods and stems to provide a whole-plant perspective on disease resistance. Finally, the correlative nature of our multi-omics data requires functional validation through techniques such as stable gene silencing, gene overexpression, or metabolic tracing to establish direct causality between gene expression, metabolite accumulation, and powdery mildew resistance. In addition, targeted quantification of key metabolites (e.g., ferulic acid and sinapic acid) using authentic standards will be necessary to validate their dynamic accumulation patterns observed in our non-targeted metabolomics analysis. Future research should also integrate proteomics to assess enzyme activities, profile root exudates and rhizosphere microbial communities to explore systemic defense connections, and conduct genome-wide association studies (GWAS) using the markers identified here. These studies will be crucial for translating these findings into durable disease resistance and sustainable pea production systems.

## 5 Conclusion

This study provides fundamental molecular and metabolic insights into the mechanisms underlying pea resistance to powdery mildew, and identifies the phenylpropanoid biosynthesis pathway as the core regulator of differential resistance between susceptible and resistant pea varieties. The distinct temporal dynamics of gene expression and metabolite accumulation between the two varieties reveal that resistant peas mount a sustained, coordinated transcriptional and metabolic defense response, while susceptible varieties exhibit a disorganized, ineffective defense that fails to contain pathogen spread. While we observed strong positive correlations between the expression of core biosynthetic genes and the accumulation of defensive metabolites for some components of the pathway, the complex, isoform-specific interplay between gene transcription and metabolite levels for other gene families underscores the intricate, multi-layered regulatory network of plant defense. The comprehensive transcriptome and metabolome datasets generated in this study serve as a critical resource for validating candidate genes and metabolites in future functional genomics studies. A deeper understanding of these defense mechanisms will be instrumental for developing more effective, disease-resistant pea varieties through molecular breeding, and for devising sustainable, eco-friendly biological control strategies for powdery mildew management.

## Data Availability

The datasets presented in this study can be found in online repositories. The names of the repository/repositories and accession number(s) can be found below: https://www.ncbi.nlm.nih.gov/, PRJNA1314297.
