# Supplementary material for: Integrated transcriptomic and metabolomic analysis reveals the central role of phenylpropanoid biosynthesis in pea resistance to powdery mildew
Source: Front Plant Sci. 2026 Apr 22;17:1793693. doi: 10.3389/fpls.2026.1793693 (PMC13144052; doi:10.3389/fpls.2026.1793693)
Supplement: Supplementary file 1 [file DataSheet1.pdf]

Supplementary Material

Supplementary Figures

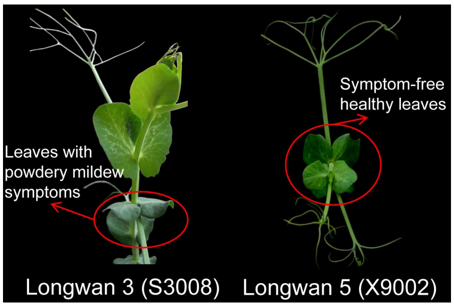

Figure S1 Typical phenotypic characteristics of powdery mildew-susceptible pea variety Longwan 3 (S3008) and resistant variety Longwan 5 (X9002) after *Erysiphe pisi* inoculation. Left panel: Longwan 3 shows typical powdery mildew symptoms on leaves; Right panel: Longwan 5 maintains healthy, symptom-free leaves, demonstrating the stable and significant difference in powdery mildew resistance between the two varieties.

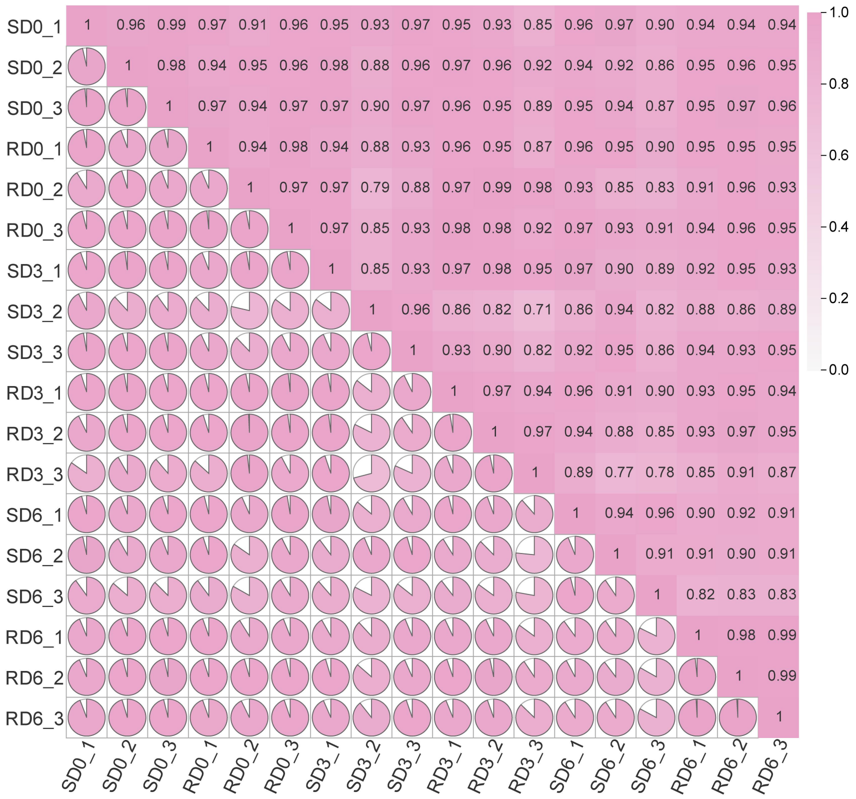

Figure S2 Correlation heatmap of transcriptomic samples of pea leaves from powdery mildew-susceptible (Longwan 3) and -resistant (Longwan 5) varieties at different days post-inoculation (dpi) (n = 3). SD0, SD3, and SD6, susceptible variety (Longwan 3) at 0, 3, and 6 dpi, respectively. RD0, RD3, and RD6, resistant variety (Longwan 5) at 0, 3, and 6 dpi, respectively.

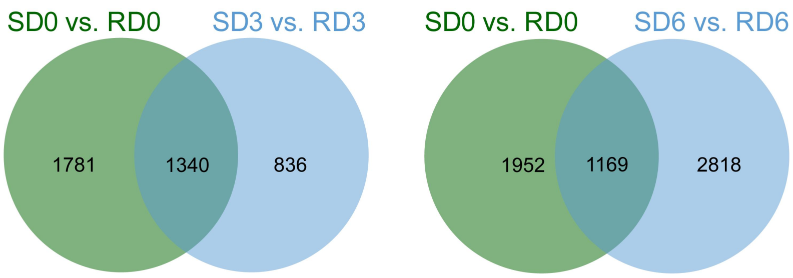

Figure S3 Venn diagram illustrating the filtering process of core resistance-related differentially expressed genes (DEGs) to eliminate inherent genetic background interference. The green circle represents the total number of DEGs identified between the susceptible and resistant varieties at pre-inoculation (SD0 vs. RD0), defined as genetic background-related DEGs unrelated to *E. pisi* infection. The blue circle represents the total number of DEGs identified between the two varieties at the corresponding infection stage. The overlapping region between the two circles indicates DEGs shared by the two comparisons, representing genetic background-related DEGs. The non-overlapping region of the blue circle represents the final core resistance-related DEGs, which are free from genetic background interference. SD0, SD3, and SD6 denote the susceptible variety (Longwan 3) at 0, 3, and 6 days post-inoculation (dpi), respectively. RD0, RD3, and RD6 denote the resistant variety (Longwan 5) at 0, 3, and 6 dpi, respectively.

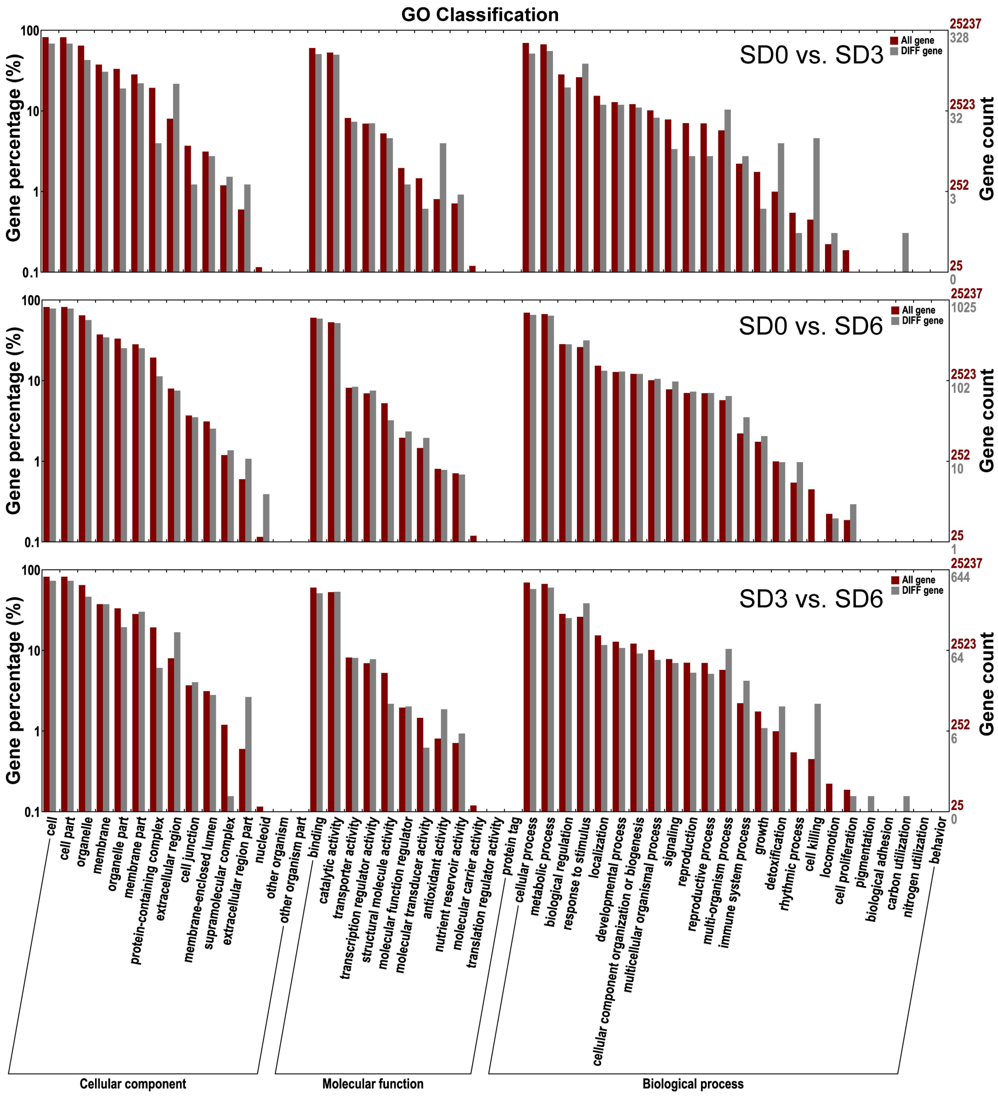

Figure S4 Gene Ontology (GO) enrichment analysis of differentially expressed genes (DEGs) in pea leaves across within-susceptible variety comparisons. SD0, SD3, and SD6, susceptible variety (Longwan 3) at 0, 3, and 6 days post-inoculation (dpi), respectively.

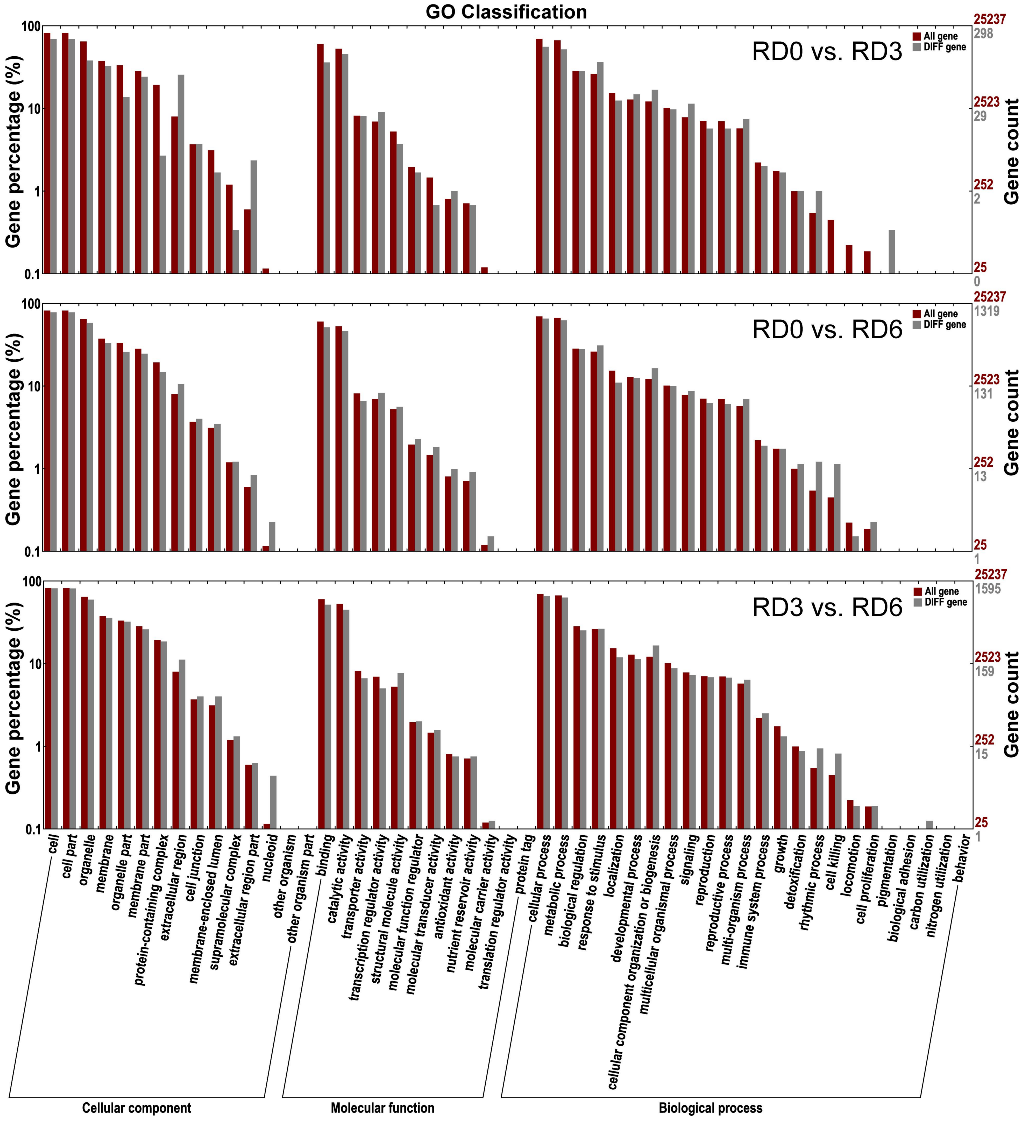

Figure S5 Gene Ontology (GO) enrichment analysis of differentially expressed genes (DEGs) in pea leaves across within-resistant variety comparisons. RD0, RD3, and RD6, resistant variety (Longwan 5) at 0, 3, and 6 days post-inoculation (dpi), respectively.

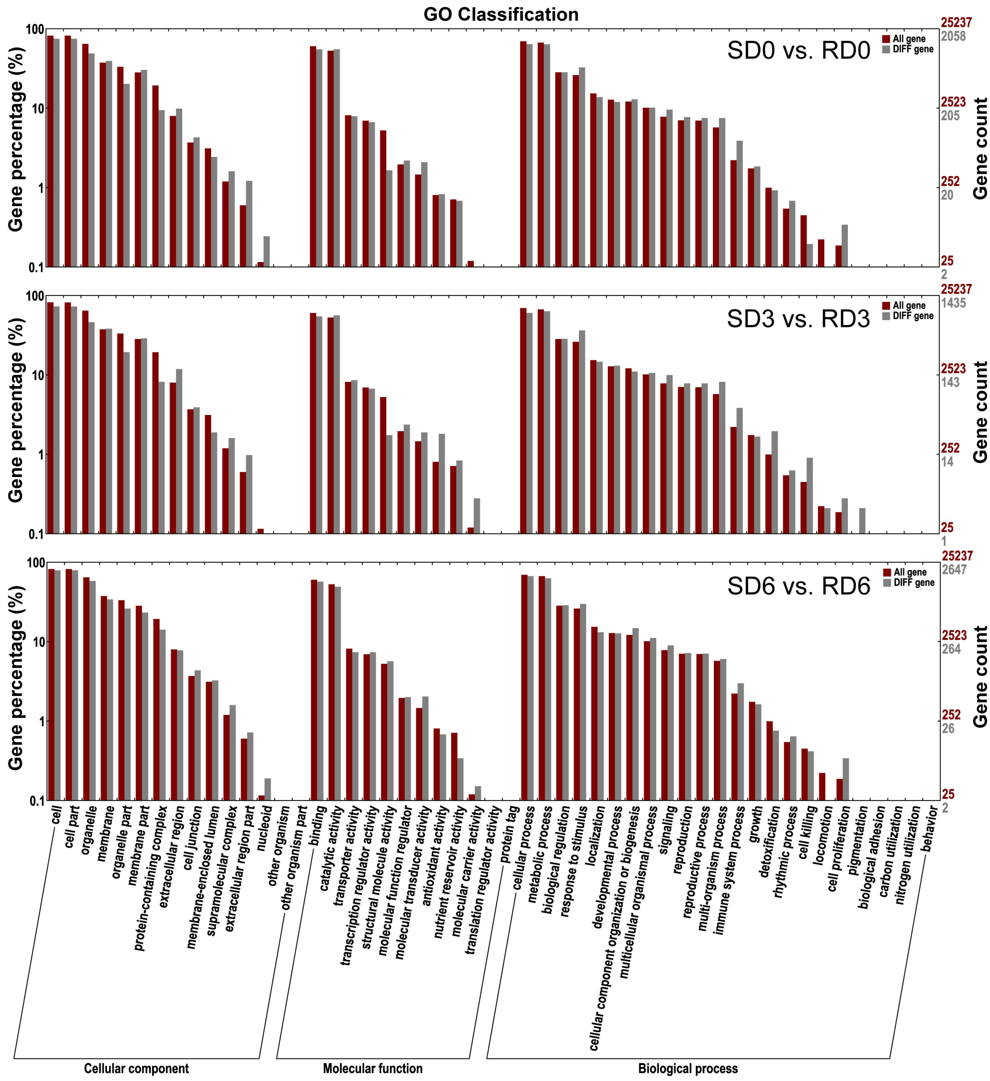

Figure S6 Gene Ontology (GO) enrichment analysis of differentially expressed genes (DEGs) in pea leaves across three pairwise comparisons. SD0, SD3, and SD6, susceptible variety (Longwan 3) at 0, 3, and 6 days post-inoculation (dpi), respectively. RD0, RD3, and RD6, resistant variety (Longwan 5) at 0, 3, and 6 dpi, respectively.

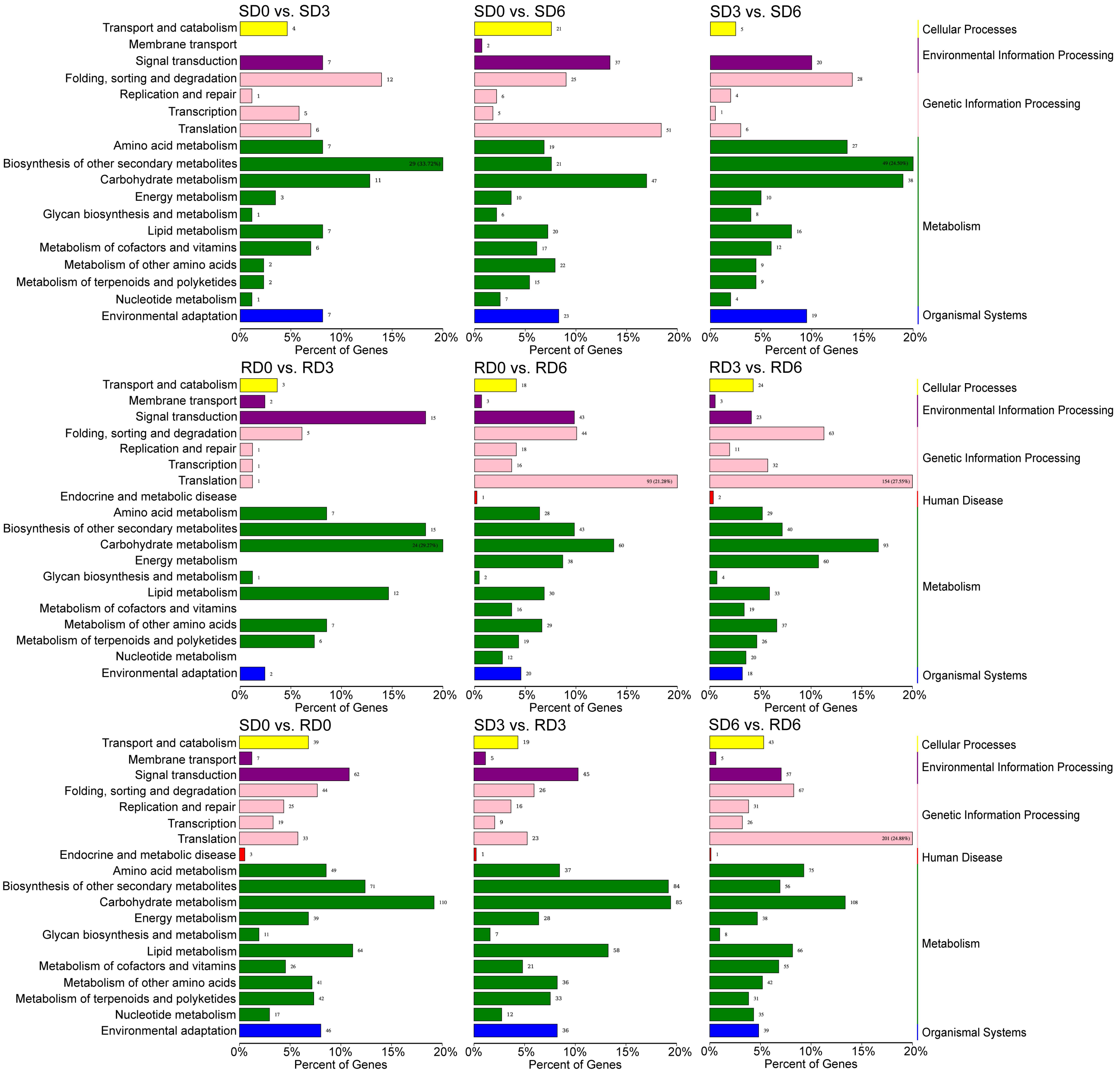

Figure S7 KEGG functional classification of differentially expressed genes (DEGs) in pea leaves across nine pairwise comparisons. SD0, SD3, and SD6, susceptible variety (Longwan 3) at 0, 3, and 6 days post-inoculation (dpi), respectively. RD0, RD3, and RD6, resistant variety (Longwan 5) at 0, 3, and 6 dpi, respectively.

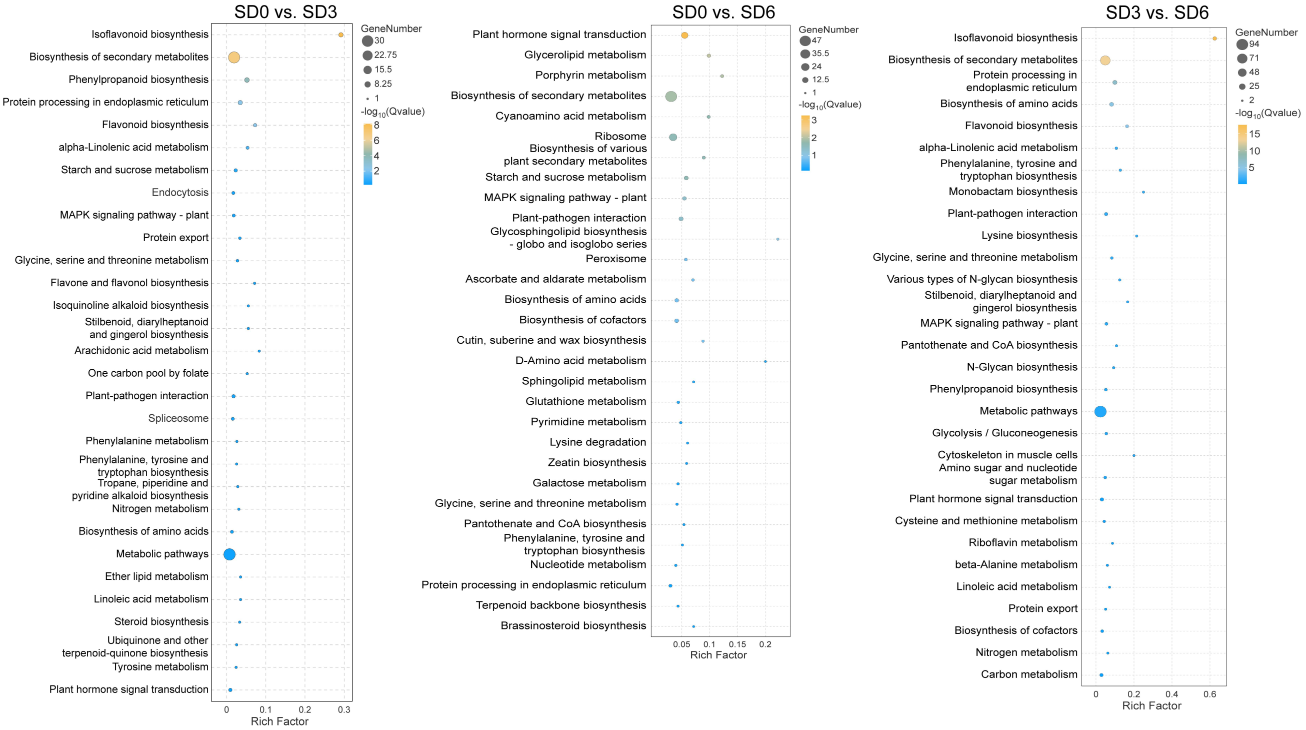

Figure S8 Bubble plot of KEGG pathway enrichment analysis of differentially expressed genes (DEGs; transcripts per kilobase million (TPM) ≥ 5) in pea leaves across within-susceptible variety comparisons. SD0, SD3, and SD6, susceptible variety (Longwan 3) at 0, 3, and 6 days post-inoculation (dpi), respectively.

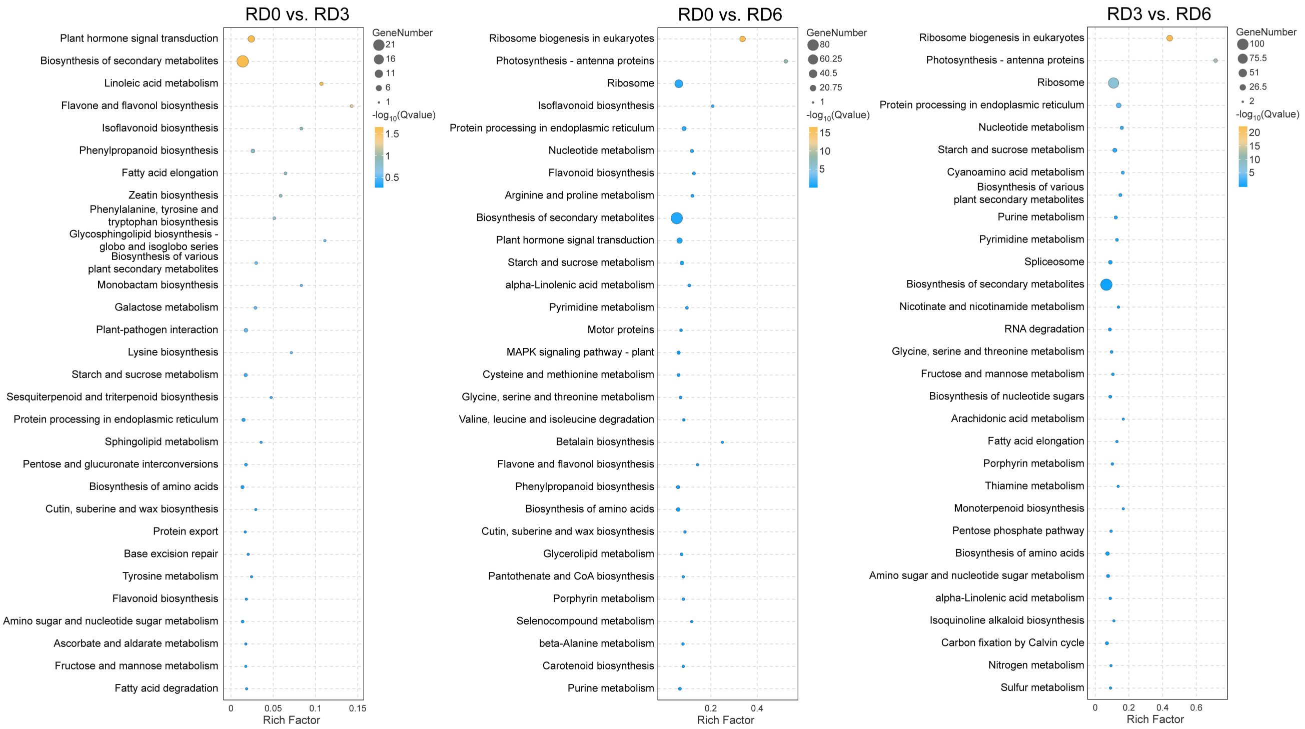

Figure S9 Bubble plot of KEGG pathway enrichment analysis of differentially expressed genes (DEGs; transcripts per kilobase million (TPM) ≥ 5) in pea leaves across within-resistant variety comparisons. RD0, RD3, and RD6, resistant variety (Longwan 5) at 0, 3, and 6 days post-inoculation (dpi), respectively.

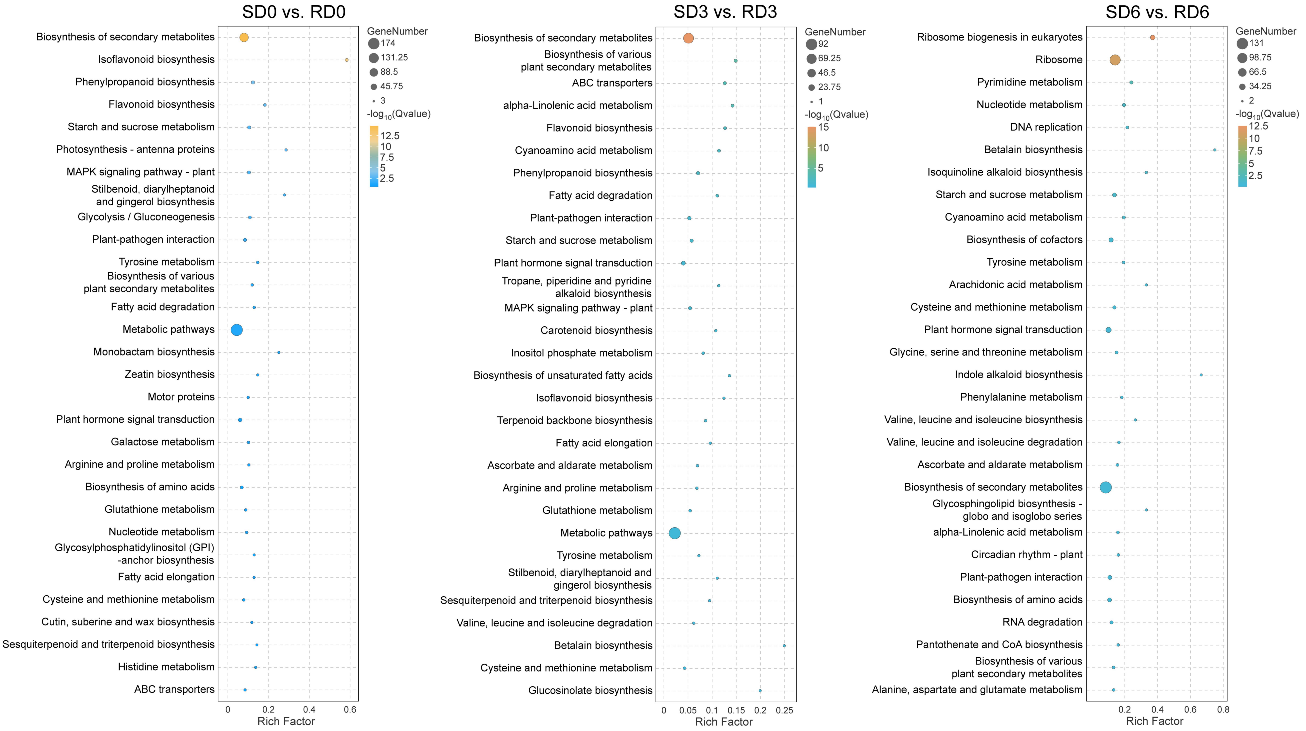

Figure S10 Bubble plot of KEGG pathway enrichment analysis of differentially expressed genes (DEGs; transcripts per kilobase million (TPM) ≥ 5) from inter-variety pairwise comparisons of pea leaves in response to *Erysiphe pisi* infection. SD0, SD3, and SD6, susceptible variety (Longwan 3) at 0, 3, and 6 days post-inoculation (dpi), respectively. RD0, RD3, and RD6, resistant variety (Longwan 5) at 0, 3, and 6 dpi, respectively.

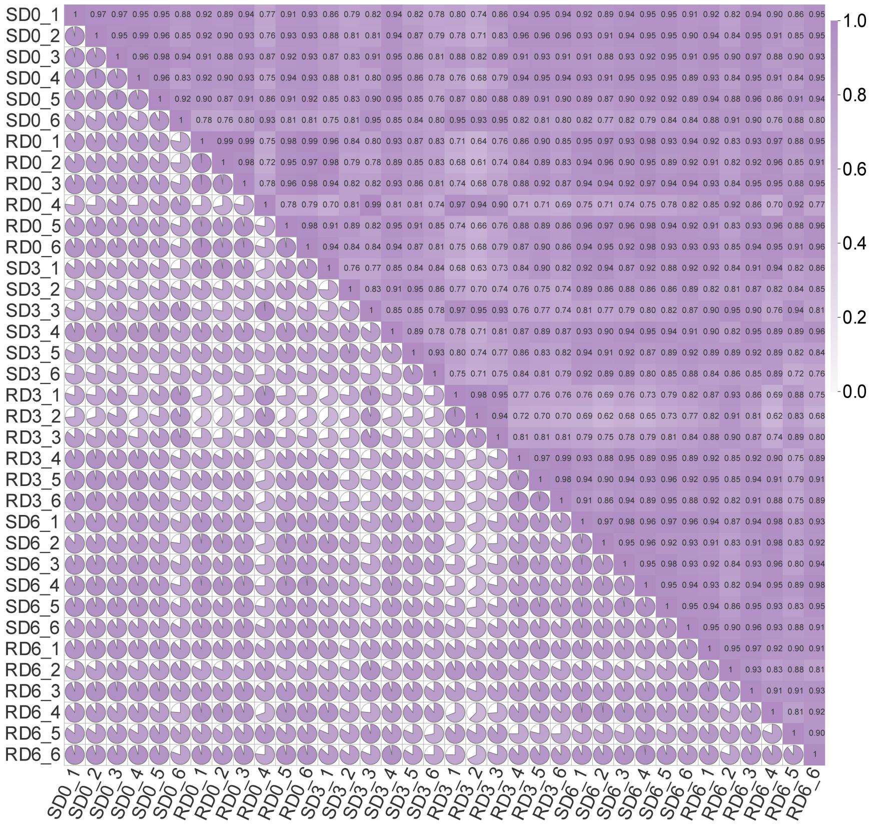

Figure S11 Correlation heatmap of metabolomic samples of pea leaves from powdery mildew-susceptible (Longwan 3) and -resistant (Longwan 5) varieties at different days post-inoculation (dpi) (n = 6). SD0, SD3, and SD6, susceptible variety (Longwan 3) at 0, 3, and 6 days post-inoculation (dpi), respectively. RD0, RD3, and RD6, resistant variety (Longwan 5) at 0, 3, and 6 dpi, respectively.

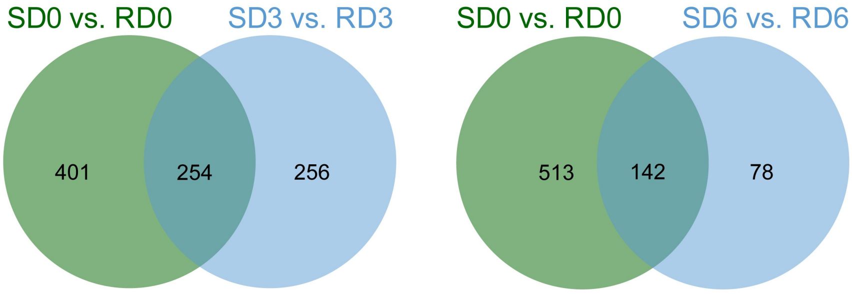

Figure S12 Venn diagram illustrating the filtering process of core resistance-related differentially accumulated metabolites (DAMs) to eliminate inherent genetic background interference. The green circle represents the total number of DAMs identified between the susceptible and resistant varieties at pre-inoculation (SD0 vs. RD0), defined as genetic background-related DAMs unrelated to *E. pisi* infection. The blue circle represents the total number of DAMs identified between the two varieties at the corresponding infection stage. The overlapping region between the two circles indicates DAMs shared by the two comparisons, representing genetic background-related DAMs. The non-overlapping region of the blue circle represents the final core resistance-related DAMs, which are free from genetic background interference. SD0, SD3, and SD6 denote the susceptible variety (Longwan 3) at 0, 3, and 6 days post-inoculation (dpi), respectively. RD0, RD3, and RD6 denote the resistant variety (Longwan 5) at 0, 3, and 6 dpi, respectively.

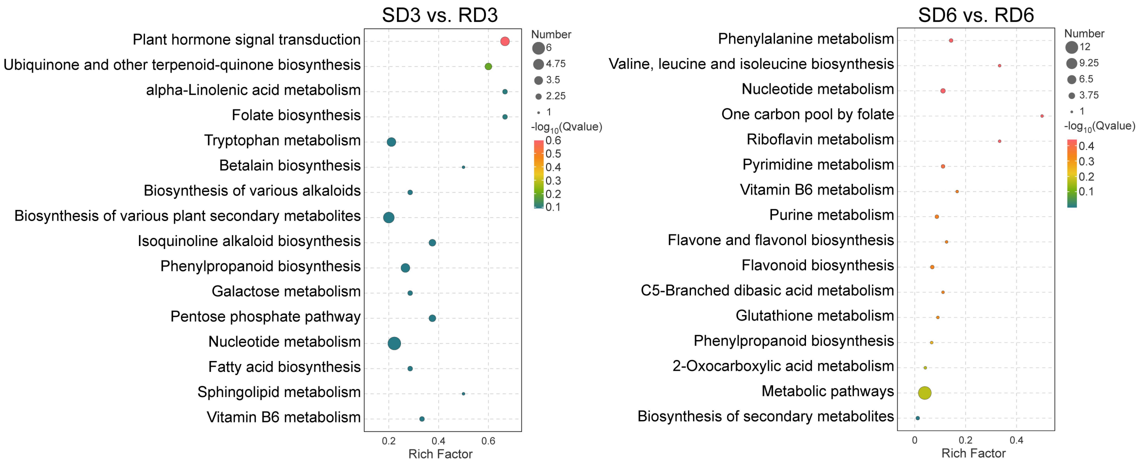

Figure S13 Bubble plot of KEGG pathway enrichment analysis of differentially accumulated metabolites (DAMs) specifically responsive to *Erysiphe pisi* inoculation, identified via intra-variety comparisons. SD3 and SD6, susceptible variety (Longwan 3) at 3 and 6 days post-inoculation (dpi). RD3 and RD6, resistant variety (Longwan 5) at 3 and 6 dpi, respectively.
